# Down regulation of Akirin-2 increases chemosensitivity in human glioblastomas more efficiently than Twist-1

**DOI:** 10.18632/oncotarget.3763

**Published:** 2015-04-18

**Authors:** Sebastian Krossa, Anne Dorothée Schmitt, Kirsten Hattermann, Jürgen Fritsch, Axel J. Scheidig, Hubertus Maximilian Mehdorn, Janka Held-Feindt

**Affiliations:** ^1^ Institute of Zoology, Department of Structural Biology, 24118 Kiel, Germany; ^2^ Department of Neurosurgery, University of Schleswig-Holstein Medical Center, 24105 Kiel, Germany; ^3^ Department of Anatomy, University of Kiel, 24118 Kiel, Germany; ^4^ Institute of Immunology, University of Schleswig-Holstein Medical Center, 24105 Kiel, Germany

**Keywords:** akirin-2, twist-1, glioblastoma, chemoresistance

## Abstract

The Twist-1 transcription factor and its interacting protein Akirin-2 regulate apoptosis. We found that in glioblastomas, highly malignant brain tumors, Akirin-2 and Twist-1 were expressed in glial fibrillary acidic protein positive tumor regions as well as in tumor endothelial cells and infiltrating macrophages / microglia. Temozolomide (TMZ) induced the expression of both molecules, partly shifting their nuclear to cytosolic localization. The knock-down (kd) of Akirin-2 increased the activity of cleaved (c)Caspase-3/-7, the amounts of cCaspases-3, -7 and cPARP-1 and resulted in an increased number of apoptotic cells after TMZ exposure. Glioblastoma cells containing decreased amounts of Akirin-2 after kd contained increased amounts of cCaspase-3 as determined by the ImageStream^x^ Mark II technology. For Twist-1, similar results were obtained with the exception that the combination of TMZ treatment and Twist-1 kd failed to significantly reduce chemoresistance compared with controls. This could be attributed to a cell population containing only slightly increased cCaspase-3 together with decreased Twist-1 levels, which was clearly larger than the respective population observed under Akirin-2 kd. Our results showed that, compared with Twist-1, Akirin-2 is the more promising target for RNAi strategies antagonizing Twist-1/Akirin-2 facilitated glioblastoma cell survival.

## INTRODUCTION

Akirins, initially discovered as genes involved in the innate immune system of *Drosophila melanogaster* [1], are a group of highly evolutionary conserved proteins among all metazoa. Knock out mutants are lethal at embryonic stage [1], and Akirins are required for NF-κB dependent gene expression in *Drosophila melanogaster* and mice [1, 2]. In vertebrates at least two genes named *akirin-1* and *akirin-2* are known [1], and in *Rattus norvegicus* Akirin-2 was described under the name FBI1 as 14-3-3β-binding protein, which acts as transcriptional repressor [3]. FBI1/Akirin-2 has been shown to be upregulated in several (rat) tumor cell lines and to promote anchorage-independent growth, tumorigenicity, and metastasis [3–5].

Nowak *et al*. [6] identified the transcription factor Twist as a novel cofactor of Akirin in *Drosophila melanogaster*, and supported a possible functional role of Akirins in tumor biology. Twist itself is an evolutionary highly conserved class B helix-loop-helix protein which e.g. is required for cranial neural tube morphogenesis or for cell survival during limb morphogenesis [7, 8]. In addition, Twist-1 is well known to be involved in cancer progression – particularly through activation of the epithelial-mesenchymal transition process which promotes tumor invasiveness and metastasis [for review: 9].

For glioblastomas (GBM), highly malignant brain tumors with a median survival of less than 15 months despite multimodal therapy [10], Twist-1 expression was detected in large amounts [11] and it contributes to glioma invasion through mesenchymal change [12]. Twist-1 confers chemoresistance to different cancers, e.g. ovarian cancer [13], cervical cancer cells [14], bladder cancer [15], and tongue squamous cell carcinoma [16]. Upregulation of Twist-1 by NF-κB blocks cytotoxicity induced by chemotherapeutic drugs [17].

Thus, both Akirin and Twist are evolutionary highly conserved nuclear proteins which seem to be involved in cancer development and progression and share some similarities like regulation of NF-κB dependent gene expression or involvement in developmental processes. Nevertheless, up to now neither the expression of Akirin in human brain tumors nor the functional role of Akirin-2 and Twist-1 in mediating chemoresistance particularly in GBMs have been investigated. Thus, the presented data focus on the expression of Akirin-2 and Twist-1 in human solid and cultured GBMs including investigations to identify the cellular assignment of these molecules. In addition, detailed analysis in which way Akirin-2 and Twist-1 confer chemoresistance to GBMs are described. In particular, transcriptional regulation of Akirin-2 and Twist-1, activated cell signaling cascades and different responses are presented for both molecules. To get an idea about possible divergent mechanistic ways, all investigations were performed in parallel with both Akirin-2 and Twist-1 in different GBM samples.

## RESULTS

### Akirin-2 and Twist-1 are expressed in human glioblastomas and are regulated through temozolomide treatment

**Figure 1:**
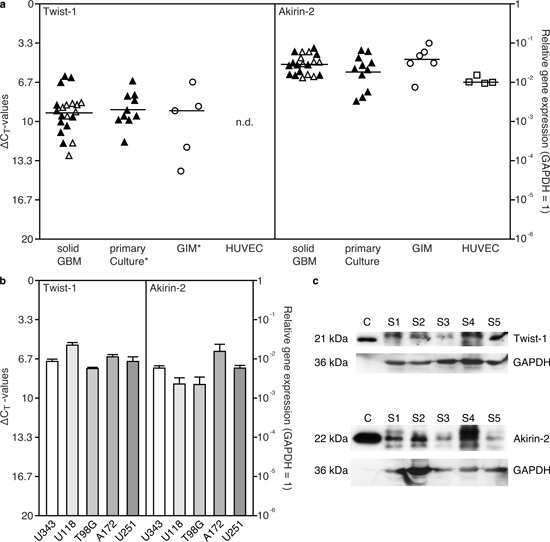
Expression of Akirin-2 and Twist-1 in a. solid and matched cultured primary human glioblastomas (GBM), glioma-infiltrating macrophages/microglia (GIM), human umbilical vein endothelial cells (HUVEC) and b. GBM cell lines was evaluated by real-time RT-PCR (measured in duplicates; logarithmic scale, ΔCT = 3.33 corresponds to a 10-fold difference; black filled symbols identify matched samples of solid and cultured GBMs), and c. Western Blot analysis of Akirin-2 and Twist-1 expression in five different solid (s) GBMs compared to recombinant Akirin-2 or Twist-1 control (c) proteins a, b. Both Akirin-2 and Twist-1 were found in distinct mRNA expression amounts in all investigated samples with Akirin-2 in comparable high level. An asterisk (*) symbolizes one Twist-1 value below detection limit in primary cultures or GIMs, respectively. c. Both Akirin-2 and Twist-1 proteins were detectable with varying amounts in different human GBM samples yielding expected sizes of 22 kDa and 21 kDa, respectively. To confirm protein integrity, blots were reprobed with glycerinaldehyde-3-phosphate-dehydrogenase (GAPDH). Representative examples of three independent experiments are shown.

Initially, we measured Akirin-2 and Twist-1 expression on mRNA and protein level in different primary human GBMs and GBM cell lines. As shown in Fig. 1, solid and cultured primary human GBMs, glioma-infiltrating macrophages/microglia (GIMs), human umbilical vein endothelial cells (HUVEC) (Fig. 1a) and five different GBM cell lines (Fig. 1b) were all characterized by distinct Twist-1 and Akirin-2 mRNA expression except for HUVEC, that did not express Twist-1. The mean Akirin-2 mRNA level was higher than the mean Twist-1 mRNA level in GIMS, solid and cultured GBMs (Fig. 1a: Twist-1 / Akirin-2 mean ΔCT values were for solid GBM samples 9.3 / 5.2, for cultured GBM cells 9.0 / 5.8 and for GIMs 9.1 / 4.7; matched samples are indicated by filled symbols) in contrast to approximately equal levels in GBM cell lines (Fig. 1b). In accordance with mRNA expression in solid GBMs, Twist-1 and Akirin-2 were detectable at variable intensities on protein level in five different solid primary human GBMs with expected sizes of 21 kDa and 22 kDa, respectively (verified by recombinant Twist-1 and Akirin-2, Fig. 1c).

**Figure 2:**
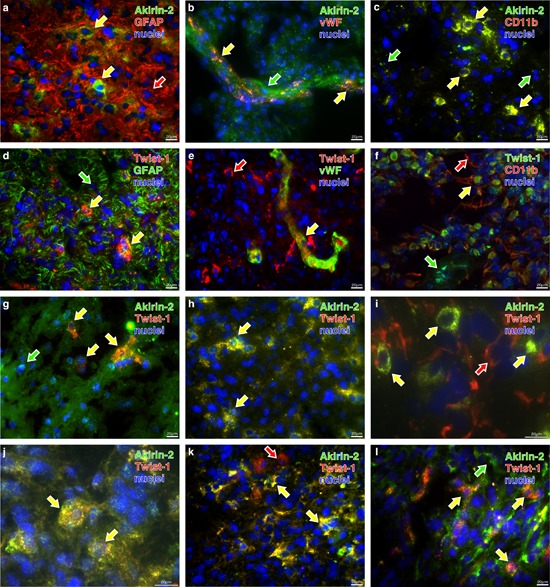
Representative costainings of Akirin-2 and Twist-1 with a-f. cellular markers (glial fibrillary acidic protein (GFAP), von Willebrand factor protein (vWF) and CD11b) and g-l. with each other in human solid GBMs as determined by immunofluorescence microscopy a-f. Akirin-2 (green) and Twist-1 (red) are found in different combinations with all cellular markers (red or green, respectively). g-l. Depending on investigated materials Akirin-2 and Twist-1 were detectable in the same tumor cell or were visible independently in different ones. Since different markers are not all localized within the same structures in the cells, signals did not merge (yellow) in all cases, but were found in the same regions. Beside double positive cells also single positive – either for stained Akirin-2 or Twist-1 or cellular marker – and double negative ones were detectable. The arrows indicate single (green or red) or double (yellow) staining of the respective markers. Magnification 400x, bar: 20 μm, representative examples of three independent experiments are shown. For individual images per dye/marker see Supplementary Figs. S1 and S2.

Double-immunofluorescence staining of solid human GBMs demonstrated that Akirin-2 and Twist-1 were found in some but not all cells in GFAP positive regions (Fig. 2a, d). Both molecules were also expressed in endothelial cells (Fig. 2b, e) and GIMs (Fig. 2c, f) present in solid GBMs. Because of different intracellular localization of the used cell type markers (GFAP, vWF, CD11b) and Twist-1/Akirin-2, signals did not merge in all cases (see also Supplementary Fig. S1). Results are in agreement with our previously performed co-stainings of Twist-1 with cell type markers (Kubelt *et al.*, in press [18]).

The co-staining of Akirin-2 and Twist-1 in several solid GBMs of different origin showed a majority of double-positive cells in most of the tumors (Fig. 2g, h, j, k) whereas in some tumors Akirin-2 and Twist-1 were mainly independently localized in different cell populations (Fig. 2i, l). Both nuclear proteins were found in cytoplasmic as well as in nuclear cellular compartments in all analyzed samples from solid human GBMs (Fig. 2 and Supplementary Fig. S2).

**Figure 3:**
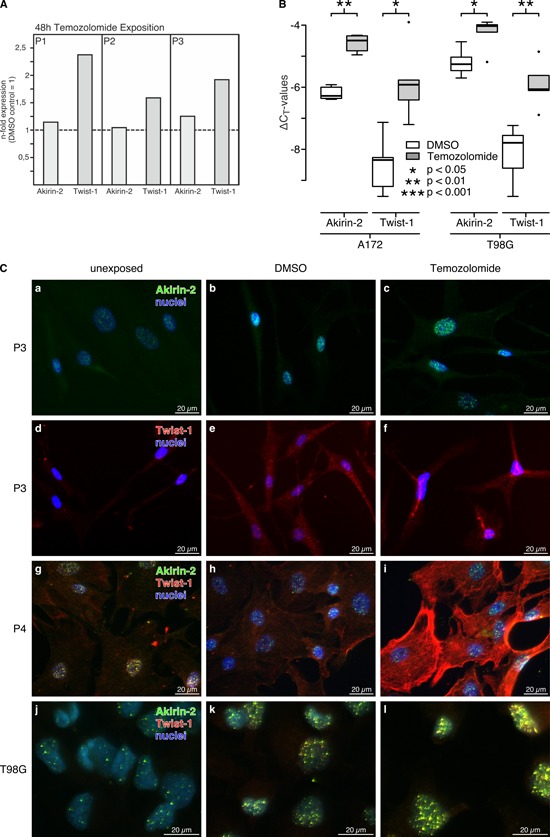
Akirin-2 and Twist-1 expression was investigated upon TMZ treatment (48 h) in A. cultured primary human GBMs (P1-P3) and B. GBM cell lines by real-time RT-PCR (logarithmic scale, ΔCT = 3.33 corresponds to a 10-fold difference; n-fold expression differences were calculated with 2^(normalized CT non-exposed – normalized CT exposed)^ = n-fold of control) In relation to dimethylsulfoxide (DMSO) TMZ clearly induced Akirin-2 and Twist-1 mRNA expression to obvious amounts in different investigated samples. Whereas primary cultures only provided enough material for individual data (performed as duplicate measurements) but results were comparable between different samples, induction of Akirin-2 and Twist-1 expression in GBM cell lines was statistically significant (n = 3; dublicates; boxplot: bold line = median; box = upper and lower quartile; whisker = 1.5-fold of interquartile range, circles = outlier). **C.** Results were confirmed by immunofluorescence staining with identical camera settings for individual experimental series. In relation to unexposed and DMSO exposed control samples for both primary cultures (P3-P4; a-i) and T98G cells **j-l**. a clear induction of protein expression of Akirin-2 and Twist-1 was found upon TMZ treatment (48 h). In primary cultures Akirin-2 seemed to be predominantly localized in the cell nucleus whereas Twist-1 was mainly found in the cytoplasm; in T98G cells both molecules were visible in the cell nucleus. Magnification 400x, bar: 20 μm, for T98G representative examples of three independent experiments are shown. For individual images per dye/marker see Supplementary Figs. S3 and S4.

As shown in Fig. 3, temozolomide (TMZ), the most common chemotherapeutic agent in GBM therapy, increased Akirin-2 and Twist-1 expression [for Twist-1 data are in accordance with our previously performed ones (Kubelt *et al.*, in press [18])]. In relation to DMSO controls, cultured human primary GBM cells (Fig. 3A) and GBM cell lines (Fig. 3B) responded to TMZ treatment with induction of Akirin-2 mRNA expression up to 1.3-fold (P3) or up to 3.4-fold (A172), and with induction of Twist-1 mRNA expression up to 2.4-fold (P1) or up to 5.4-fold (A172), respectively. In comparison with Akirin-2, the Twist-1 induction appeared stronger but also wider distributed in all analyzed cells. Comparable results were observed on protein level (Fig. 3C) for both cultured human primary GBM (Fig 3C, a-i) and T98G cells (Fig. 3C, j-l). Of note, Twist-1 and Akirin-2 levels were increased after DMSO exposure alone, which can induce apoptosis under certain conditions [19, 20], and further increased after TMZ exposure compared with unexposed controls (Fig. 3C a vs. b/c; d vs. e/f; g vs. h/i; j vs. k/l). Additionally, slight mRNA expression changes, especially for Akirin-2, resulted in a distinct increase of Akirin-2 in the nucleus or Twist-1 in the cytosol, respectively (matched cultured GBM cells P3, Fig. 3A and 3C, a-f). In contrast to the solid GBM samples and their heterogeneous nature, in primary cultures, Akirin-2 seemed to be predominantly localized in the cell nucleus whereas Twist-1 was mainly found in the cytoplasm (Fig. 3C, a-i and Supplementary Figs. S3 and S4a-i). The GBM cell line T98G exclusively contained detectable amounts of both proteins in the cell nucleus (Fig. 3C, j-l and Supplementary Fig. S4j-r).

### Akirin-2 and Twist-1 confer chemoresistance to human glioblastomas

Since TMZ exposure significantly increased expression of Akirin-2 and Twist-1 on mRNA and protein level, we speculated that both molecules are involved in processes which contribute to chemoresistance in human GBM cells. Thus, we analyzed the effects of siRNA knock-down (kd) of Akirin-2 and Twist-1 on TMZ-induced apoptosis.

**Figure 4:**
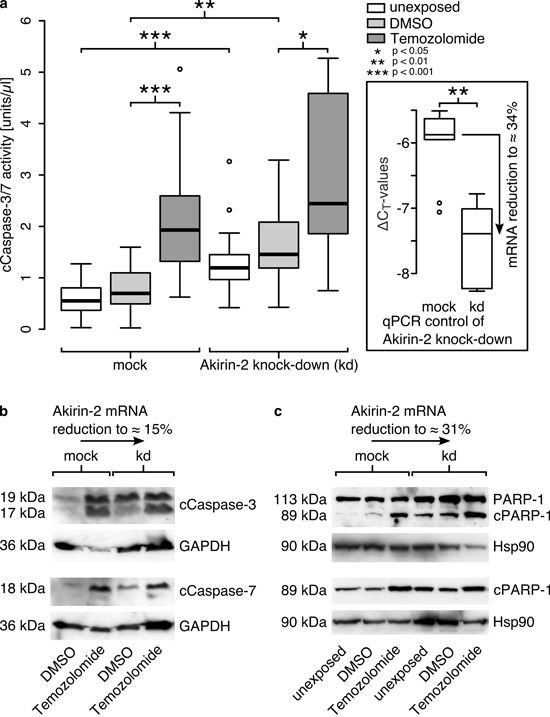
Akirin-2 mediated chemoresistance could be abolished by RNAi technology as measured by (a) cleaved (c)Caspase-3/-7 activity assay, (b) cCaspase-3 and -7 and (c) cleaved (c)poly(ADP-ribose) polymerase-1 (cPARP-1) Western Blots **a.** In relation to mock transfected T98G cells Akirin-2 knock down (kd) was able to induce cell death to significantly greater extents in dimethylsulfoxide (DMSO) as well as in TMZ (400 μg/ml, 24 h) treated samples (n = 6; triple values; boxplot: bold line = median; box = upper and lower quartile; whisker = 1.5-fold of interquartile range, circles = outlier). **b.** Results were confirmed by Western Blot using specific antibodies directed against cCaspase-3 and -7, respectively. c. PARP-1 Western Blot using two different antibodies specifically directed against cleaved PARP-1 (bottom) or both uncleaved and cleaved PARP-1 (top) additionally approved results. Equal protein loading was confirmed by detection of glycerinaldehyde-3-phosphate-dehydrogenase (GAPDH) or heat shock protein 90 (Hsp90), and efficiency of knock down was proven by qRT-PCR for all experiments in parallel. Representative examples of two independent experiments are shown.

In relation to mock transfected controls, Akirin-2 kd resulted in higher cleaved Caspase-3/-7 (cCaspase-3/-7) activity in all analyzed samples. Of note, the cCaspase-3/-7 activity was already significantly increased in unexposed and DMSO exposed control cells [Fig. 4a; median for: unexposed cells: 0.55 U/μl (mock), 1.19 U/μl (kd); DMSO exposed cells: 0.70 U/μl (mock), 1.46 U/μl (kd); TMZ treated cells: 1.93 U/μl (mock), 2.44 U/μl (kd)]. The efficiency of each Akirin-2 kd was proven by qRT-PCR (Akirin-2 mRNA reduction to approximately 15–34%, Fig. 4). As described above, DMSO and TMZ exposure only moderately increased Akirin-2 mRNA levels, but led to a clearly visible accumulation of Akirin-2 in the nucleus (Fig. 3). Thus, this DMSO- and TMZ-mediated inducible effect on Akirin-2 expression most likely did not abolish but partially bypassed and counteracted the Akirin-2 siRNA kd and thereby probably led to heterogeneously responding cell (sub-) populations. This can explain the observed higher cCapsase-3/-7 activity with a much broader, asymmetric distribution in TMZ exposed cells after Akirin-2 kd. Nevertheless, a clearly larger upper quartile (Fig. 4a, upper box of TMZ exposed mock vs. kd) shows the strong tendency towards higher cCaspase-3/-7 activity after Akirin-2 kd. The effect of the Akirin-2 kd to increase the basal cCaspase-3/-7 activity in GBM cells and to sensitize the cells to DMSO and TMZ exposure was further supported by Western Blot (WB) experiments. The cCaspase-3 and -7 were detected in higher amounts in siAkirin-2 kd cells than in mock cells after DMSO exposure (Fig. 4b). In addition, cleaved PARP-1 (cPARP-1), which is involved in a number of cellular processes including DNA repair and cell death, was found in higher amounts in Akirin-2 kd than in mock transfected GBM cells, unexposed or exposed to DMSO (Fig. 4c). After TMZ exposure, due to maximal signal intensities already seen in mock controls, the performed WB experiments did not allow differentiation of cCaspase-3/-7 and cPARP-1 amounts between Akirin-2 kd and mock GBM cells after TMZ exposure any more. Nevertheless, when counting apoptotic cells based on chromatin condensation beside its effect on basal and DMSO exposed cells also an induction of apoptosis after TMZ exposure was detectable, supporting our previous findings (amount of apoptotic cells [mean ± standard deviation]: unexposed 15 ± 5 % mock vs. 22 ± 6 % kd; DMSO exposed 9 ± 2 % mock vs. 26 ± 8 % kd; TMZ exposed 40 ± 8 % mock vs. 69 ± 10 % kd). Akirin-2 kd consistently and clearly increased the number of apoptotic cells after DMSO and TMZ exposure compared with mock cells. Summarized, these results emphasize Akirin-2 as an attenuator of pro-apoptotic signals thereby conferring chemoresistance against apoptosis inducers like TMZ.

Contrary to expectations, the Twist-1 kd mediated effects on cCaspase-3/-7 activities and amounts as well as on cPARP-1 amounts were unreliable (data not shown). Whereas in some experiments clearly anti-apoptotic effects of Twist-1 were measurable, in others Twist-1 kd yielded a better survival of treated GBM cells. In accordance with this, counting of apoptotic cells after Twist-1 kd and TMZ exposure indicated a less prominent effect compared to Akirin-2 kd (amount of apoptotic cells [mean ± standard deviation]: unexposed 18 ± 4% mock vs. 25 ± 6% kd; DMSO exposed 18 ± 2% mock vs. 30 ± 6% kd; TMZ exposed 28 ± 6% mock vs. 36 ± 8% kd). These results were obtained despite the fact that Twist-1 kd was effective, as proven by qRT-PCR.

**Figure 5:**
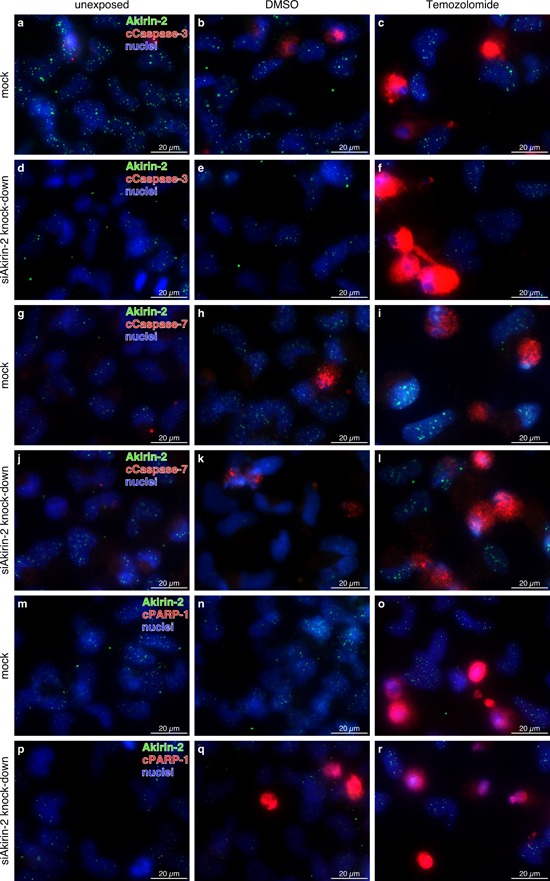
Representative costainings of Akirin-2 (green) with a-f. cleaved (c)Caspase-3, g-l. cleaved (c)Caspase-7, and (m-r) cleaved (c)poly(ADP-ribose) polymerase-1 (cPARP-1) (all red) in unexposed, dimethylsulfoxide (DMSO) or TMZ (400 μg/ml, 24 h) treated mock or siAkirin-2 transfected T98G GBM cells Although no clear quantitative data could be obtained, in relation to mock transfected samples (a-c, g-I, m-o) Akirin-2 knock down (d-f, j-l, p-r) resulted in higher amounts of cCaspase-3, -7 or cPARP-1 positively stained cells especially in DMSO and TMZ treated samples. Irrespective of treatment, Akirin-2 knock down became visible by lower intensities and amounts of Akirin-2 positively stained cells. Magnification 400x, bar: 20 μm, representative examples of two independent experiments are shown. For individual images per dye/marker see Supplementary Figs. S5, S6 and S7.

**Figure 6:**
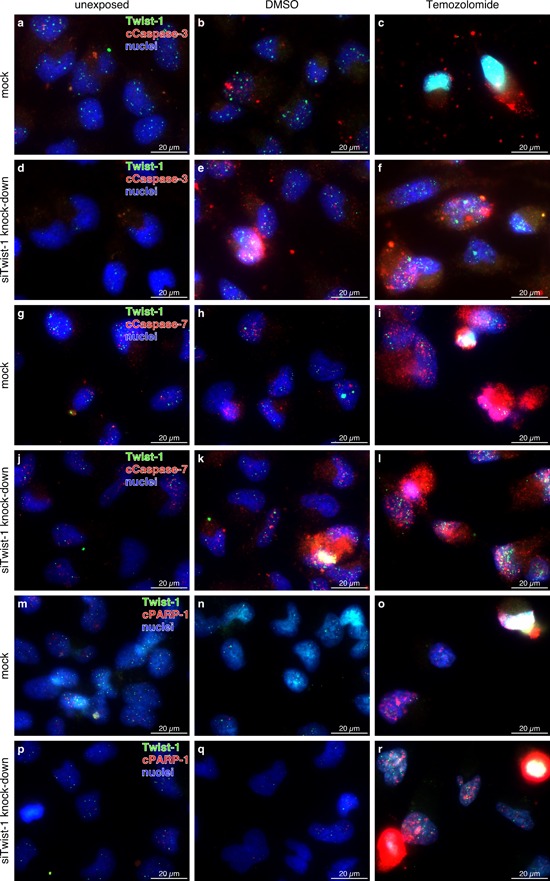
Representative costainings of Twist-1 (green) with a-f. cleaved (c)Caspase-3, g-l. cleaved (c)Caspase-7, and m-r. cleaved (c)poly(ADP-ribose) polymerase-1 (cPARP-1) (all red) in unexposed, dimethylsulfoxide (DMSO) or TMZ (400 μg/ml, 24 h) treated mock or siTwist-1 transfected T98G GBM cells. Although clear quantitative data could not be obtained, in relation to mock transfected samples (a-b, g-h, m-n) Twist-1 knock down (d-e, j-k, p-q) resulted in higher amounts of cCaspase-3, -7 or cPARP-1 positively stained cells in unexposed and DMSO treated samples, respectively, whereas in TMZ treated ones (c vs. f, i vs. l, o vs. r) no distinct differences were detectable Twist-1 knock down became visible by lower intensities and amounts of Twist-1 positively stained cells in unexposed and DMSO treated samples, with TMZ application Twist-1 knock down seemed to be antagonized. Magnification 400x, bar: 20 μm, representative examples of two independent experiments are shown. For individual images per dye/marker see Supplementary Figs. S8, S9 and S10.

The cCaspase-3/-7 activity assays and Western Blot techniques deal with total cell/protein amounts. It is not possible to analyze potential variations in single cells or cell subpopulations in different samples with these assays, which might be the critical point for unreliable Twist-1 results. Thus, we decided to analyze the effects of both Akirin-2 and Twist-1 kd in unexposed, DMSO and TMZ treated cells with a different, single cell-based approach. For this purpose we performed a double-immunofluorescence staining of mock and Akirin-2 kd or Twist-1 kd TMZ exposed cells. To allow for comparisons with previous investigations, staining of Akirin-2 or Twist-1 in combination with cCaspase-3 and -7 as well as with cPARP-1 was realized (Fig. 5 for Akirin-2, Fig. 6 for Twist-1 results). With this approach quantitative data could not be generated, but it was sufficient to give a first hint which changes may occur on single cell level.

For Akirin-2, results were consistent with results described above. The amount of cells positive for the different apoptosis-associated proteins increased after DMSO and TMZ exposure in all immunofluorescence stainings. As hypothesized above, we found a heterogeneous response of different cell (sub) populations to TMZ exposure. Besides cells that contained high amounts of cCaspase-3, -7 or cPARP-1, cells containing none or only low amounts of these proteins were detected under DMSO or TMZ exposure (Fig. 5). Cells double positive for, Akirin-2 and cCaspase-3, -7 or cPARP1, as well as cells only positive for cCaspase-3, -7 or cPARP1 were detected (Fig 5 and Supplementary Figs. S5, S6 and S7). Thereby, double-staining showed an effective Akirin-2 reduction on the protein level (Fig. 5 and Supplementary Figs. S5, S6 and S7, compare mock and Akirin-2 kd rows), and minor staining intensities of Akirin-2 were combined with higher staining intensities for different cell death associated molecules (Fig. 5 and Supplementary Figs. S5, S6 and S7; DMSO or TMZ column). In addition, although based on these stainings not quanttification of the difference of the response of mock and Akirin-2 kd cells to TMZ (and DMSO) exposure can be obtained, inspection of additional images (data not shown) indicated a tendency towards an increased amount of apoptosis-associated protein positive cells after Akirin-2 kd. All camera settings were identical for individual experimental series and efficiency of Akirin-2 kd, proven by qRT-PCR in parallel, yielded a reduction of Akirin-2 mRNA up to 27%.

Accordingly, double-immunofluorescence staining of Twist-1 together with cCaspase-3, -7 or cPARP-1 revealed an influence on cell death of this nuclear protein. Under DMSO and TMZ exposure the amount of cells that were positively stained for one of the apoptosis-associated proteins increased. The heterogeneous response to TMZ exposure, described above, was confirmed. Cells double positive for Twist-1 and cCaspase-3, -7 or cPARP1 as well as single positive ones were detected. Cells with a strong staining intensity of apoptosis-associated proteins mostly contained a low staining intensity for Twist-1 and *vice versa*. Nevertheless, individual cells with strong staining intensities for Twist-1 and cCaspase-3, -7 or cPARP1 were observed (Fig. 6 and Supplementary Figs. S8, S9 and S10). But, despite the fact that no quantitative data could be obtained with the used approach, when comparing mock and RNAi transfected TMZ treated cells it seemed that the difference in induction of cell death was nearly equal (Fig. 6 and Supplementary Figs. S8, S9 and S10; TMZ column). Additionally, in contrast to cells with lower Twist-1 signal intensities in siTwist-1 transfected unexposed and DMSO exposed samples (compared to mock, mRNA reduction up to 29%), in TMZ treated ones, some cells were visible with nearly similar staining intensities of Twist-1 in mock and RNAi transfected cells (Fig. 6 and Supplementary Figs. S8, S9 and S10, compare mock and Akirin-2 kd rows).

To analyze the described effect in more detailed and quantitative manner we continued our investigations using an ImageStream^x^ Mark II analysis. This “high resolution on flow” method combines flow cytometry with the functional insights of high resolution microscopy to give a detailed insightful cell analysis. Thus, we anticipated to get a better idea about amounts of cCaspase-3, Akirin-2 and Twist-1 double and single positively stained cells and portions of Akirin-2 and Twist-1 quantities in TMZ (un-) exposed mock and RNAi transfected cells, respectively.

### Three different Akirin-2 or Twist-1 / cCaspase-3 double positively stained cell populations exist

**Figure 7:**
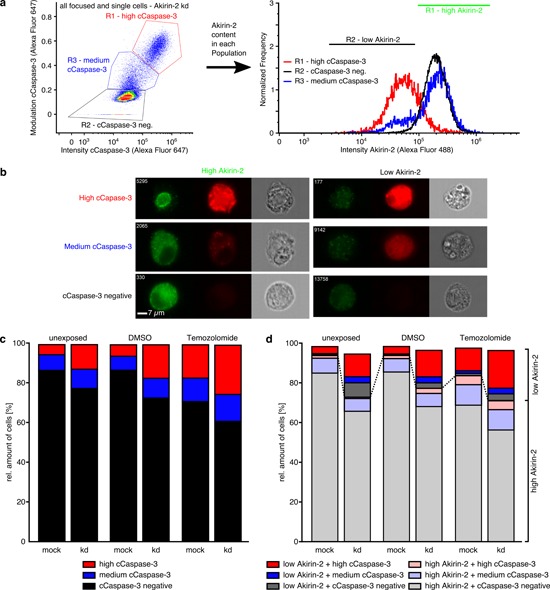
ImageStream^x^ Mark II analysis of costainings of Akirin-2 (green) with cleaved (c)Caspase-3 (far red) in unexposed, dimethylsulfoxide (DMSO) or TMZ (400μg/ml, 24 h) treated mock or siAkirin-2 knock down (kd) T98G GBM cells **a, b.** A high, medium and a cCaspase-3 negative cell population with variable Akirin-2 contents was detectable in all analyzed samples, and **c.** amounts of high cCaspase-3 positively stained cells increased from unexposed up to TMZ treated samples with higher amounts in siAkirin-2 transfected cells. **d.** Higher contents of a low Akirin-2 + high cCaspase-3 positive cell population were found for siAkirin-2 transfected cells in unexposed, DMSO and TMZ treated samples. Representative examples of two independent experiments are shown.

**Figure 8:**
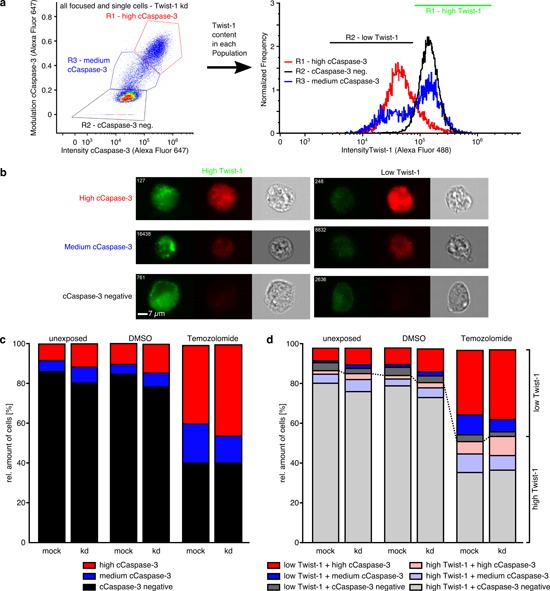
ImageStream^x^ Mark II analysis of costainings of Twist-1 (green) with cleaved (c)Caspase-3 (far red) in unexposed, dimethylsulfoxide (DMSO) or TMZ (400 μg/ml, 24 h) treated mock or siTwist-1 transfected T98G GBM cells **a-b.** A high, medium and a cCaspase-3 negative cell population with variable Twist-1 contents was detectable in all analyzed samples, and **c.** amounts of high cCaspase-3 positively stained cells increased from unexposed up to DMSO treated samples with higher amounts in siAkirin-2 transfected cells. In TMZ treated samples summarized amounts of high and medium cCaspase-3 positively stained T98 cells were nearly equal including **d.** a prominent low Twist-1 + medium cCaspase-3 cell population resulting in obliteration of differences between mock and RNAi samples. Representative examples of two independent experiments are shown.

We observed, that in all samples three individual cell populations, based on the cCaspase-3 staining, were distinguishable for all unexposed, DMSO and TMZ exposed mock, siAkirin-2 and siTwist-1 transfected cells, respectively (Figs. 7, 8). Here, only cells inside the focus of the microscope (no blurry cells) and only single cells (no doublets, triplets) were included in the analysis.

The combined information of total cCaspase-3 staining intensity and the contrast and texture of the cCaspase-3 staining (represented by the modulation value) per single cell resulted in clearly distinguishable populations: cCaspase-3 negative and high (intensity/modulation) cCaspase-3 positive cells (black and red boxes in the left plot in Figs. 7a, 8a). In addition, cells with an approximately equal to slightly higher total intensity and a higher modulation compared with the cCaspase-3 negative population and a slightly lesser total intensity and lower modulation compared with the high (intensity/modulation) cCaspase-3 population were detected (blue boxes in the left plot in Figs. 7a, 8a). Together with the manual inspection of randomly sampled cell images (representative examples in Fig. 7b, 8b), we defined the thresholds for in total three populations, introducing a medium (intensity/modulation) cCaspase-3 population.

The amounts of high and medium cCaspase-3 positively stained cells increased from unexposed up to TMZ treated samples and were found in higher amounts in siAkirin-2 transfected cells compared with mock cells (Fig. 7c). In addition, all three cell populations were characterized by different Akirin-2 contents (Fig. 7a, b) with low Akirin-2 amounts mostly found in high cCaspase-3 populations and high Akirin-2 amounts predominantly detectable in cCaspase-3 negative populations (Fig. 7a, d). Thus, a reciprocal association of Akirin-2 and cCaspase-3 within the same GBM cell was visible, and kd of Akirin-2 (mRNA reduction up to 18%) clearly induced higher contents of cCaspase-3. Although comparable effects were observed for Twist-1 stained cells (Fig. 8), in contrast to Akirin-2 kd, the medium and high cCaspase-3 population appeared quantitatively more prominent after TMZ treatment (Fig. 8d). In detail, especially a much higher amount of cells in the low Twist-1 + medium cCaspase-3 population was detectable (e.g. mock: 10% in relation to Akirin-2 samples with 0.3%), and this low Twist-1 + medium cCaspase-3 fraction was higher in mock than in siTwist-1 (mRNA reduction up to 30%) treated cells after TMZ application (Fig. 8d). In comparison, slightly lower to equal amounts of low Twist-1 + medium cCaspase3 positive cells were measurable in unexposed or DMSO treated Twist-1 samples (Fig. 8c and d). Thus, despite the fact that in relation to Akirin-2 kd after Twist-1 kd a higher amount of high cCaspase-3 cells (32.4 % vs 3.8 %) was detectable, the medium cCaspase-3 population equalized the amount of cells with detectable cCaspase-3 levels between mock and siTwist-1 treated cells under TMZ application (Fig. 8c and d).

Summarized, we were able to show that both nuclear proteins Akirin-2 and Twist-1 are expressed in human primary GBMs on mRNA and protein level, and are induced upon TMZ treatment, with Twist-1 to greater extents. When comparing Akirin-2 and Twist-1 effects in mediating chemoresistance to GBMs, it became obvious that RNAi strategies modulated the distribution of cells between the three observed cell populations (characterized by cCaspase-3 levels). Treatment with siAkirin-2 sensitized cells to TMZ induced cell death whereas no distinct effect of Twist-1 RNAi could be seen. This could be attributed to both a strong Twist-1 induction through TMZ application which partly antagonizes RNAi strategy, and a prominent low Twist-1 + medium cCaspase-3 population. This distinct low Twist-1 / medium cCaspase-3 cell population which obliterated differences between mock and RNAi samples might represent cells responding to TMZ exposition with Twist-1 expression and thereby antagonizing the cCaspase-3 induction. This assumption would explain the observed lower amount of low Twist-1 + medium cCaspase-3 cells after siTwist-1 treatment under TMZ application. Thus, here we present completely new insight on the role of Akirin-2 and Twist-1 in mediating chemoresistance in GBM cells, and show that Akirin-2 seems to be the more promising target when using RNAi strategies to antagonize Akirin-2 mediated GBM cell survival.

## DISCUSSION

In 2012, Nowak and colleagues published interesting results concerning an interaction of the nuclear factors Akirin and Twist in *Drosophila melanogaster* [6]. Using a yeast double-interaction screen, they found that, mechanistically, Akirin mediates a novel connection between Twist and a chromatin remodeling complex to facilitate changes in the chromatin environment, leading to the optimal expression of some Twist-regulated genes during *Drosophila melanogaster* myogenesis [6]. Thus, Akirin seems to be a secondary cofactor that serves as an interface between a critical developmental transcription factor (like Twist) and the chromatin remodeling machinery [21]. Complementary, since Twist-1 is well known in mediating progression of various tumors, an involvement of Akirin-2 in tumor progression seems to be rather likely.

Beside others, one main characteristic of tumor progression is the marked chemoresistance of malignant entities. For Twist-1 some groups were able to show its influence on mediating chemoresistance [13–17, 22, 23]. For GBMs, highly malignant brain tumors with profound chemoresistance, a possible role of Twist-1 in mediating this aspect is still not investigated. In addition, Akirin-2 expression and functional role in GBMs are completely unknown.

Here we now showed for the first time that Akirin-2 is expressed in human primary glioblastomas on mRNA and protein level, and is induced upon TMZ treatment. Established Twist-1 expression in GBMs [12, 24] could be also confirmed in our system and additionally we were able to show that TMZ treatment induced Twist-1 expression to large extents. These results are in accordance with currently unpublished data of our group concerning expression and regulation of different epithelial-to-mesenchymal transition markers, including Twist-1, in matched pairs of primary and recurrent human GBMs. Additionally, here we were able to show that Akirin-2 kd by RNAi led to decreased chemoresistance in GBMs generating three different cell populations defined by varying amounts of Akirin-2 and cCaspase-3. In contrast, upon TMZ treatment, a potential Twist-1 facilitated chemoresistance could not be crucially influenced by siTwist-1 strategy. Since efficiency of Twist-1 knock down was verified both on mRNA and protein levels (qRT-PCR, immunocytochemistry and low Twist-1 group in ImageStream analysis) this could be attributed to both a strong Twist-1 induction which partly antagonizes RNAi strategy and to a distinct low Twist-1 + medium cCaspase-3 cell population which obliterated differences between mock and RNAi samples.

For Akirin-2, our results are in line with previously published ones which demonstrated that the rat Akirin-2 homolog FBI1 promotes tumorigenicity and metastasis of Lewis lung carcinoma cells [4], and acts as a transcriptional repressor promoting anchorage-independent growth [3]. In addition, investigations by Akiyama et al. [5] showed that the basal cell adhesion molecule (BCAM), an immunoglobulin superfamily membrane protein that acts as a laminin α5 receptor, seems to be a FBI1/Akirin-2 target gene in rats. Hereby, a 14-3-3β-FBI1/Akirin-2 complex binds to the BCAM promotor and represses transcription of BCAM resulting in downregulation of this suppressive oncogene [5]. Thus, a tumor-promoting function of Akirin-2 is obvious and now clearly addressed also in the human system.

For Twist-1, presented data do not contradict others showing that Twist-1 confers chemoresistance to different cancers, e.g. ovarian cancer [13], bladder cancer [15], and tongue squamous cell carcinoma [16]. Upregulation of Twist-1 by NF-κB blocks cytotoxicity induced by chemotherapeutic drugs [17], and Twist-1 inhibits the induction of p53-mediated apoptosis in rodent fibroblasts in response to genotoxins and prolonged serum deprivation suggesting that Twist-1 could function as an oncogene [23]. Whereas in TMZ treated GBMs Twist-1 RNAi could not significantly reduce chemoresistance, short interfering RNA directed against Twist-1 increases non-small cell lung cancer sensitivity to cisplatin via the MAPK/mitochondrial pathway [22] and short hairpin RNA targeting Twist-1 suppressed cell proliferation and improves chemosensitivity to cisplatin in HeLa human cervical cancer cells [14]. Thus, it seems that under diverse chemotherapeutic treatment strategies and when investigating variable tumor entities Twist-1-dependend chemoresistance differed in response to antagonizing strategies. This effect becomes more important when antagonizing strategies modulate the distribution of individual cell populations which are characterized by variable efficiency in mediating cell death. Interestingly, Zhu et al. [14] were able to show that shTwist-1 transfection generated both early and late apoptotic cells. Nevertheless, in mentioned examples no clear analysis were performed if knock down strategies generated different cell populations with variable efficiency in mediating tumor cell death.

Taken together, here we described completely new data to understand the role of Akirin-2 and Twist-1 in the context of chemoresistance in GBM cells, and showed that Akirin-2 seems to be the more promising molecule when using RNAi strategies for antagonizing Akirin-2 triggered GBM cell survival.

## MATERIALS AND METHODS

### Cell lines and tumor specimens

The human GBM cell lines U343, U251 (formerly known as U373), A172, U118 and T98G were obtained from the DKFZ (Heidelberg, Germany), and human umbilical vein endothelial cells (HUVEC) were purchased from PromoCell (Heidelberg, Germany). Solid and cultured human GBM samples were freshly surgical dissected tissues from the Department of Neurosurgery (Kiel, Germany) and were obtained in accordance with the Helsinki Declaration of 1975 with approval of the ethics committee of the University of Kiel, Germany (file reference: D 485/12) after written consent of donors. The diagnosis was established by a pathologist, and tumors were classified according to the WHO criteria. When sufficient material was available, matched samples of solid / cultured primary human GBMs were used for different experiments.

### Isolation of GIMs, cell culture of primary human GBM cells

In order to isolate glioma infiltrating macrophages/microglia (GIMs) freshly obtained human GBM samples were subjected to CD11b MACS separation. Therefore, single-cell suspensions of tumor tissue were generated using the Neural Dissociation Kit (T) (Miltenyi Biotech GmbH, Bergisch Gladbach, Germany), and cells were immediately labeled with CD11b MicroBeads (Miltenyi Biotech GmbH) and separated using MACS LS columns according to the manufacturer's instructions and as described before [25]. Cultured primary human GBM cells were generated by dissociation and cultivation in DMEM (Invitrogen, Karlsruhe, Germany) plus 10% fetal calf serum (FCS; Invitrogen) as previously described [25, 26]. The different GBM cells were checked for purity by immunostaining with cell type-specific markers and for the absence of Mycoplasma contamination by staining with bisbenzimide as described before [25, 26].

### Real-time RT-PCR (qRT-PCR)

RNA was isolated from temozolomide (TMZ) exposed/unexposed glioma cell lines (see below) and cultured primary human GBM cells, solid GBMs and from GIMs and HUVEC with the TRIZOL reagent (Invitrogen), digested by DNase, cDNA was synthesized, and quantitative real time RT-PCR (qRT-PCR) was performed [27, 28] using TaqMan primer probes (Applied Biosystems, Foster City, CA, USA): glycerinaldehyde-3-phosphate-dehydrogenase (*hGAPDH*; Hs99999905_m1)*, hAkirin-2* (Hs00363236_m1), *hTwist-1 (*Hs01675818_s1). The reaction was carried out with the MyiQ^TM^ Single Color Real-time PCR Detection System (BIO-RAD, München, Germany) and fluorescent data were converted into cycle threshold (C_T_) measurements. ΔC_T_ values of each sample were calculated as CT_gene of interest_ – CT _GAPDH._ Relative gene expression was calculated with 2^(normalized CT non-exposed – normalized CT exposed)^ = n-fold of control. A ΔC_T_ value of 3.33 corresponds to one magnitude lower gene expression compared to GAPDH. For each gene, logarithmic linear dependence of C_T_-values from the numbers of copies was verified by using different amounts of cDNA.

### Western blot

Solid human GBMs and transfected/TMZ exposed T98G cells (see below) were harvested with lysis buffer, up to 25 μg of protein per sample was loaded on 10% (anti-cleaved PARP-1, anti-PARP-1) or 18% (anti-Twist-1, anti-Akirin-2, anti-cleaved Caspase-3, and -7) SDS-polyacrylamide gels for electrophoresis and then transferred to a polyvinylidene difluoride membrane (Hybond^TM^-P PVDF membrane, GE Healthcare, Freiburg, Germany) as previously described [29]. After incubation with 2% casein/TBST, the membranes were incubated with primary antibodies against anti-Akirin-2 (HS00055122-MO1; 1:250; Abnova, Heidelberg, Germany), anti-Twist-1 (Hs00007291-MO3; 1:250; Abnova), anti-cleaved(c)PARP-1 (poly(ADP-ribose) polymerase; #9541; 1:250; Cell Signaling Technology, Boston, MA, USA), anti-PARP-1 (#9532; 1:250; Cell Signaling Technology), anti-cCaspase-3 (#9661; 1:160; Cell Signaling Technology), and anti-cCaspase-7 (#8438; 1:200; Cell Signaling Technology), respectively. The membranes were incubated with the secondary antibody (1:20,000, donkey anti-rabbit IgG-HRP or 1:50,000, donkey-anti mouse IgG-HRP; Santa Cruz Biotechnology; Heidelberg, Germany), and horseradish peroxidase activity was detected by applying an ECL Advance Western Blotting Detection Kit (GE Healthcare) followed by exposure of the membranes to a sheet of autoradiography film (Hyperfilm^TM^ECL^TM^, GE Healthcare). Recombinant Akirin-2 and Twist-1 (synthetic cDNA obtained from Genscript, Piscataway, USA) were co-expressed as 6xHis-GST-fusion proteins in E. coli BL21(DE3) strains using a modified (two equal expression cassettes containing Akirin-2 and Twist-1 cDNA, respectively) pETM33 plasmid (EMBL, Heidelberg, Germany). Cells were cultivated in 1 l LB media in 5 l baffled Erlenmeyer flasks at 37°C in a standard lab shaking incubator inoculated with an overnight culture to a starting absorbance at 600 nm (OD600) of approx. 0.05. Induction occurred at an OD600 of approx. 0.6 with 1 mM IPTG. Cells were harvested 3 h after induction by centrifugation (10 min, 7000 g, 4°C). Resuspended cells (1:5 (w:v) ratio in 50 mM bis-tris-propane pH 9, 500 mM NaCl, 1 mM dithiothreitol (DTT), 10% (v/v) glycerol ( = standard buffer) supplemented with 10 mM imidazole and 1 mM phenylmethanesulfonyl fluoride (PMSF)) were desintegrated by two passages through an Emulsiflex C3 (Avestin, Ottawa, Canada) with pulses of 1200 bar. After two subsequent centrifugation steps (30 min, 75600 g, 4°C), the proteins were simultaneously purified via the N-terminal 6xHis-tag using a HisTrap HP 5 ml column and an ÄKTA Purifier FPLC system (both GE Healthcare). Proteins were bound to the equilibrated column (10 column volumes (CV) of standard buffer) followed by a washing step of 10 CV (50 mM imidazole in standard buffer) and a linear elution gradient over 10 CV to a final concentration of 500 mM imidazole in standard buffer. The 6xHis-GST-tag was separated from both proteins via overnight incubation at 4°C with human rhinovirus 3C protease (1:100 (w:w) ratio) in fresh standard buffer. Buffer was exchanged according to manufacturer recommended standard procedure with HiTrap Desalting 5 ml (GE Healthcare) columns. Resulting samples served as positive control for Akirin-2 and Twist-1 Western Blot experiments. Equal protein loading and protein integrity were confirmed by either reprobing the membranes with anti-GAPDH (sc-20357; 1:300; Santa Cruz Biotechnology) or anti-HSP90 (H-114; 1:1,000; Santa Cruz Biotechnology) after antibody stripping using Reblot Stripping Solution (Millipore, Temecula, CA, USA).

### Immunofluorescence – solid materials

Cryostat sections of different GBM tissues were fixed in acetone/methanol, washed with Tris-buffered saline plus Tween, blocked with Sudan black, rinsed with ethanol until dye free, blocked with bovine serum albumin and glycine in TBS, and then without washing incubated with primary antibodies in TBS-T as described before [30, 31]. Since all primary antibodies were obtained from mouse, incubation with the first primary and secondary antibody-pair was followed by a blocking step with donkey anti-mouse FAB-fragments (1:1000; Dianova, Hamburg, Germany) before the second pair of primary and secondary antibodies was applied. Primary antibodies were omitted for negative controls, and incubations were performed in darkness to maintain the fluorescence of the secondary antibodies. Nuclei were stained with 4´, 6-diamidino-2-phenylindole (DAPI; Invitrogen; 1:30000). After embedding in Immu-Mount (Shandon, Pittsburgh, PA, USA) digital photography was performed using a Zeiss microscope and Zeiss camera (Zeiss, Oberkochem, Germany).

In combination with anti-glial fibrillary acidic protein (GFAP) (MAB3402; 1:800; mouse monoclonal; Millipore), anti-von Willebrand factor protein (vWF) (sc-53465; 1:1000; mouse monoclonal, Santa Cruz Biotechnology), and anti-CD11b (sc-1186; 1:250; mouse monoclonal, Santa Cruz), the antibodies anti-Twist-1 (H00007291-M03; 1:500; mouse monoclonal, Abnova), anti-Akirin-2 (HS00055122-MO1; 1:500; Abnova) were stained first either with Alexa Fluor 488 or Alexa Fluor 555 coupled secondary antibodies (green or red, 1:1000, donkey anti-mouse IgG, Invitrogen), the second secondary antibody detecting the cellular markers was either donkey anti-mouse IgG Alexa Fluor 555 or 488 (red or green, 1:1000, Invitrogen).

### Exposure to temozolomide of non-transfected cultured human glioma cells

Cultured primary human GBM cells as well as glioma cell lines (1.0 - 2.0 × 10^5^ cells) were exposed to 400 μg/ml TMZ (Sigma-Aldrich, Taufkirchen, Germany) in DMEM supplemented with 0.5% FCS for up to 48 h. Corresponding controls were unexposed or exposed to an equal volume (in regard to TMZ) of dimethylsulfoxide (DMSO) in DMEM supplemented with 0.5% FCS for up to 48 h. Afterwards RNA was isolated using TRIZOL reagent (described above), or cultured cells were used for immunofluorescence staining (see below).

### Immunofluorescence – cultured cells

Both transfected/TMZ exposed (see below) or only TMZ exposed cultured primary human GBM cells, glioma cell lines and corresponding controls were grown on sterile glass cover slips in 10% FCS-supplemented DMEM, and fixed with methanol-acetone (1:1; ice-cold) for 10 min. Non-specific binding was blocked with 0.5% bovine serum albumin (BSA) / 0.5% glycine in PBS for 60 min. Glass cover slips were incubated with anti-Akirin-2 (HS00055122-MO1; 1:500; Abnova) or anti-Twist-1 (H00007291-M03; 1:500; mouse monoclonal, Abnova) as first primary antibody over night at 4°C. The primary antibody was omitted for negative controls. Glass cover slips were incubated with the Alexa Fluor 488 or 555 coupled first secondary antibody (green or red, 1:1000, donkey anti-mouse IgG, Invitrogen) for 1 h at 37°C. If both primary antibodies were from mouse an additional blocking step with donkey anti-mouse FAB-fragments (1:1000; Dianova) was performed. The second primary antibody - for transfected/TMZ exposed cells and corresponding controls anti-cPARP (#9541; 1:100; Cell Signaling Technology) or anti-cCaspase-3 (#9661; 1:200, Cell Signaling Technology) or anti-cCaspase-7 (#8438; 1:200, Cell Signaling Technology), for only TMZ exposed cells and corresponding controls *vice versa* anti-Akirin-2 or anti-Twist-1 - was applied over night at 4°C. Cells were incubated with Alexa Fluor 488 or 555 coupled second secondary antibody (green or red, 1:1000, donkey anti-rabbit or donkey anti-mouse IgG, Invitrogen). Nuclei were stained with DAPI (Invitrogen; 1:30000; 30 min room temperature). After embedding in Immu-Mount (Shandon), digital photography was performed using a Zeiss fluorescence microscope and Zeiss camera (Zeiss). To obtain comparable results between single approaches, all microscope and camera settings were identical for individual experimental series.

### RNAi silencing

After cultivation of T98G glioma cells in DMEM plus 10% FCS in 6-well dishes (180,000 cells / well; for Western Blot experiments and ImageStream^x^ Mark II analysis), 96-well dishes (15,000 cells / well; for caspase-3/7 activity assay) or on sterile glass cover slips (15,000 cells / well; for immunofluorescence staining) for 24 h, cells were transfected with siTwist-1 RNA (siRNA ID: 4390824 ; 60 pmol / well; Life Technologies, Darmstadt, Germany), transferred to a mixture of Opti-MEM Medium and lipofectamine (Life Technologies) for 6 h, or with siAkirin-2 (siRNA ID: D-00102-00002; 50 nmol / well; riboxx Life Sciences, Radebeul, Germany) transferred to a mixture of Opti-MEM Medium and riboxx®FECT (riboxx Life Sciences) for 24 h. In parallel a transfection with silencer select negative control siRNA (Life Technologies) or negative control pool (riboxx Life Science) was performed under same conditions. Cells were washed 20 min for three times, respectively, with DMEM plus 0.5 % FCS and afterwards exposed to 400 μg/ml TMZ, unexposed or exposed to an equal volume of DMSO for 24 h and applied for immunofluorescence staining, Western Blot, Caspase-Assay or ImageStream^x^ Mark II Analysis as described above/ below, respectively.

To control the knock down efficiency, RNA of transfected cells was purified in parallel with the PicoPure RNA Isolation Kit (MDS Analytical Technologies, Sunnyvale, CA) according to the manufacturer's instructions, and qRT-PCR using TaqMan primer probes (Applied Biosystems): *hGAPDH* (Hs99999905_m1)*, hAkirin-2* (Hs00363236_m1), and *hTwist-1 (*Hs01675818_s1) was performed as described above.

### cCaspase-3/7 activity assay

After RNAi silencing and TMZ exposure of T98G cells (described before) samples were washed in PBS and incubated in 100μl Homogeneous cCaspase-3/7 substrate (Apo-ONE® Homogeneous Caspase-3/7 Assay; Promega, Madison, USA) for 30 min according to the manufacturer's instruction and as described before [27]. The amounts of active Caspase-3/7 were determined in relation to a Caspase-7 standard (Enzo Life Science, Lörrach, Germany) as units/μl.

### Determination of the amount of apoptotic cells in adherent cell culture

RNAi-silencend and TMZ exposed T98G cells were grown in duplicates on sterile glass cover slips, stained with DAPI and subsequently microscopic images were taken from randomly chosen locations. Counting of at least 1,000 cells per sample was performed by two independent and blinded persons. Non-apoptotic and apoptotic cells were distinguished by nuclear fragmentation. The arithmetic mean and the standard deviation of individual countings were calculated with Microsoft Excel 2007.

### ImageStream^x^ Mark II analysis

After RNAi silencing and TMZ exposure of T98G cells (described before) both harvested adherent and detached, suspended cells were collected, fixed with ice-cold 2% paraformaldehyde / PBS for 15 min, permeabilized with ice-old 0.2% saponine / 0.1% BSA / PBS for 15 min, washed with PBS, blocked with ice-cold 1% BSA / PBS for 15 min, and incubated with a mixture of primary antibodies including anti-Twist-1 (H00007291-M03; 1:500; Abnova), anti-Akirin-2 (HS00055122-MO1; 1:500; Abnova) and anti-cCaspase-3 (#9661; 1:200; Cell Signaling Technology) in 1% BSA / PBS for 60 min on ice. Afterwards, cells were washed, incubated with a mixture of Alexa Fluor 488 or 647 coupled secondary antibodies (green or far-red, 1:1000, donkey anti-rabbit or donkey anti-mouse IgG, Invitrogen) in 1% BSA / PBS for 60 min on ice. After a final washing step with PBS, cells were resuspended in 20–30 μl PBS and ImageStream^x^ Mark II analysis was performed. For each sample 10,000 cells were measured. Data for channel compensation for each dye was collected from single stained samples. Collected data were analyzed with the IDEAS software (Amnis Seattle, USA). Gates for focused and single cells were set according to the manufacturer's recommendations. Initially, three populations were equally defined for all measured samples based on distribution of cCaspase-3 signal intensity vs. signal modulation (normalized intensity range of an image, modulation = [max pixel – min pixel]/[max pixel + min pixel]). Subsequently, two populations were equally defined for all measured samples based on distribution of Akirin-2 or Twist-1 signal intensity and applied to the three cCaspase populations. All graphs (dot-plot, histograms and cell images) and statistics (cell count and relative amount) were generated with the IDEAS software. Statistics were visualized with Microsoft Excel 2007.

### Statistical analysis

Statistical test and box-and-whisker plots generation were performed using the software R (version 2.14.1). Data were tested for normal distribution with the Kolmogorov-Smirnov test. Due to non-normal distribution, all data were subsequently tested with the nonparametric Wilcoxon rank sum test.

## SUPPLEMENTARY FIGURES



## Abbreviations

BSA: bovine serum albumin
C_T_: cycle of threshold
DMEM: Dulbecco's modified Eagle's medium
DMSO: dimethylsulfoxide
FCS: fetal calf serum
GAPDH: glycerinaldehyde-3-phosphate-dehydrogenase
GBM: glioblastoma
GFAP: glial fibrillary acidic protein
GIM: glioma infiltrating macrophages/microglia
HUVEC: human umbilical vein endothelial cells
kd: knock down
PARP: poly(ADP-ribose) polymerase
PBS: phosphate buffered saline
qRT-PCR: quantitative reverse-transcription polymerase chain reaction
TMZ: temozolomide
vWF: von Willebrand factor protein
WHO: World Health Organization

## References

[1] Goto A, Matsushita K, El Chamy L, Gesellchen V, Kuttenkeuler D, Takeuchi O, Hoffmann JA, Akira S, Boutros M (2008). Akirins are highly conserved nuclear proteins required for NF-kappaB-dependent gene expression in drosophila and mice. Nature immunology.

[2] Macqueen DJ, Johnston IA (2009). Evolution of the multifaceted eukaryotic akirin gene family. BMC evolutionary biology.

[3] Komiya Y, Kurabe N, Katagiri K, Ogawa M, Sugiyama A, Kawasaki Y, Tashiro F (2008). A novel binding factor of 14-3-3beta functions as a transcriptional repressor and promotes anchorage-independent growth, tumorigenicity, and metastasis. The Journal of biological chemistry.

[4] Komiya Y, Akiyama H, Sakumoto R, Tashiro F (2014). FBI1/Akirin2 promotes tumorigenicity and metastasis of Lewis lung carcinoma cells. Biochem Bioph Res Co.

[5] Akiyama H, Iwahana Y, Suda M, Yoshimura A, Kogai H, Nagashima A, Ohtsuka H, Komiya Y, Tashiro F (2013). The FBI1/Akirin2 Target Gene, BCAM, Acts as a Suppressive Oncogene. Plos One.

[6] Nowak SJ, Aihara H, Gonzalez K, Nibu Y, Baylies MK (2012). Akirin Links Twist-Regulated Transcription with the Brahma Chromatin Remodeling Complex during Embryogenesis. Plos Genet.

[7] Chen ZF, Behringer RR (1995). Twist Is Required in Head Mesenchyme for Cranial Neural-Tube Morphogenesis. Gene Dev.

[8] Zuniga A, Quillet R, Perrin-Schmitt F, Zeller R (2002). Mouse Twist is required for fibroblast growth factor-mediated epithelial-mesenchymal signalling and cell survival during limb morphogenesis. Mech Develop.

[9] Sanchez-Tillo E, Liu YQ, de Barrios O, Siles L, Fanlo L, Cuatrecasas M, Darling DS, Dean DC, Castells A, Postigo A (2012). EMT-activating transcription factors in cancer: beyond EMT and tumor invasiveness. Cell Mol Life Sci.

[10] Ohgaki H, Kleihues P (2005). Epidemiology and etiology of gliomas. Acta Neuropathol.

[11] Elias MC, Tozer KR, Silber JR, Mikheeva S, Deng M, Morrison RS, Manning TC, Silbergeld DL, Glackin CA, Reh TA, Rostomily RC (2005). TWIST is expressed in human gliomas and promotes invasion. Neoplasia.

[12] Mikheeva SA, Mikheev AM, Petit A, Beyer R, Oxford RG, Khorasani L, Maxwell JP, Glackin CA, Wakimoto H, Gonzalez-Herrero I, Sanchez-Garcia I, Silber JR, Horner PJ, Rostomily RC (2010). TWIST1 promotes invasion through mesenchymal change in human glioblastoma. Mol Cancer.

[13] Nuti SV, Mor G, Li P, Yin G (2014). TWIST and ovarian cancer stem cells: implications for chemoresistance and metastasis. Oncotarget.

[14] Zhu KX, Chen LH, Han XB, Wang J, Wang J (2012). Short hairpin RNA targeting Twist1 suppresses cell proliferation and improves chemosensitivity to cisplatin in HeLa human cervical cancer cells. Oncol Rep.

[15] Chen YL, Li L, Zeng J, Wu KJ, Zhou JC, Guo P, Zhang D, Xue Y, Liang L, Wang XY, Chang LS, He DL (2012). Twist Confers Chemoresistance to Anthracyclines in Bladder Cancer through Upregulating P-Glycoprotein. Chemotherapy.

[16] Liu M, Wang JG, Huang HZ, Hou JS, Zhang B, Wang AX (2013). miR-181a-Twist1 pathway in the chemoresistance of tongue squamous cell carcinoma. Biochem Bioph Res Co.

[17] Pham CG, Bubici C, Zazzeroni F, Knabb JR, Papa S, Kuntzen C, Franzoso G (2007). Upregulation of Twist-1 by NF-kappa B blocks cytotoxicity induced by chemotherapeutic drugs. Mol Cell Biol.

[18] Kubelt C, Hattermann K, Sebens S, Mehdorn HM, Held-Feindt J Epithelial-to-mensenchymal transition in paired human primary and recurrent glioblastomas. Int J Oncol.

[19] Yuan C, Gao JY, Guo JC, Bai L, Marshall C, Cai ZY, Wang LM, Xiao M (2014). Dimethyl Sulfoxide Damages Mitochondrial Integrity and Membrane Potential in Cultured Astrocytes. Plos One.

[20] Hanslick JL, Lau K, Noguchi KK, Olney JW, Zorumski CF, Mennerick S, Farber NB (2009). Dimethyl sulfoxide (DMSO) produces widespread apoptosis in the developing central nervous system. Neurobiol Dis.

[21] Nowak SJ, Baylies MK (2012). Akirin: a context-dependent link between transcription and chromatin remodeling. Bioarchitecture.

[22] Zhuo WL, Wang Y, Zhuo XL, Zhang YS, Chen ZT (2008). Short interfering RNA directed against TWIST, a novel zinc finger transcription factor, increases A549 cell sensitivity to cisplatin via MAPK/mitochondrial pathway. Biochem Bioph Res Co.

[23] Maestro R, Dei Tos AP, Hamamori Y, Krasnokutsky S, Sartorelli V, Kedes L, Doglioni C, Beach DH, Hannon GJ (1999). twist is a potential oncogene that inhibits apoptosis. Gene Dev.

[24] Velpula KK, Dasari VR, Tsung AJ, Dinh DH, Rao JS (2011). Cord blood stem cells revert glioma stem cell EMT by down regulating transcriptional activation of Sox2 and Twist1. Oncotarget.

[25] Held-Feindt J, Hattermann K, Muerkoster SS, Wedderkopp H, Knerlich-Lukoschus F, Ungefroren H, Mehdorn HM, Mentlein R (2010). CX3CR1 promotes recruitment of human glioma-infiltrating microglia/macrophages (GIMs). Exp Cell Res.

[26] Hattermann K, Held-Feindt J, Lucius R, Muerkoster SS, Penfold MET, Schall TJ, Mentlein R (2010). The Chemokine Receptor CXCR7 Is Highly Expressed in Human Glioma Cells and Mediates Antiapoptotic Effects. Cancer Res.

[27] Tong Y, Mentlein R, Buhl R, Hugo HH, Krause J, Mehdorn HM, Held-Feindt J (2007). Overexpression of midkine contributes to anti-apoptotic effects in human meningiomas. J Neurochem.

[28] Mentlein R, Forstreuter F, Mehdorn HM, Held-Feindt J (2004). Functional significance of vascular endothelial growth factor receptor expression on human glioma cells. J Neuro-Oncol.

[29] Held-Feindt J, Rehmke B, Mentlein R, Hattermann K, Knerlich F, Hugo HH, Ludwig A, Mehdorn HM (2008). Overexpression of CXCL16 and its receptor CXCR6/bonzo promotes growth of human schwannomas. Glia.

[30] Li G, Hattermann K, Mentlein R, Mehdorn HM, Held-Feindt J (2013). The transmembrane chemokines CXCL16 and CX3CL1 and their receptors are expressed in human meningiomas. Oncol Rep.

[31] Hattermann K, Li G, Hugo HH, Mentlein R, Mehdorn HM, Held-Feindt J (2013). Expression of the chemokines CXCL12 and CX3CL1 and their receptors in human nerve sheath tumors. Histol Histopathol.

