# Supplementary material for: Down regulation of Akirin-2 increases chemosensitivity in human glioblastomas more efficiently than Twist-1
Source: Oncotarget. 2015 Apr 18;6(25):21029–45. doi: 10.18632/oncotarget.3763 (PMC4673248; doi:10.18632/oncotarget.3763)
Supplement: Supplementary file 1 [file oncotarget-06-21029-s001.pdf]

SUPPLEMENTARY FIGURES

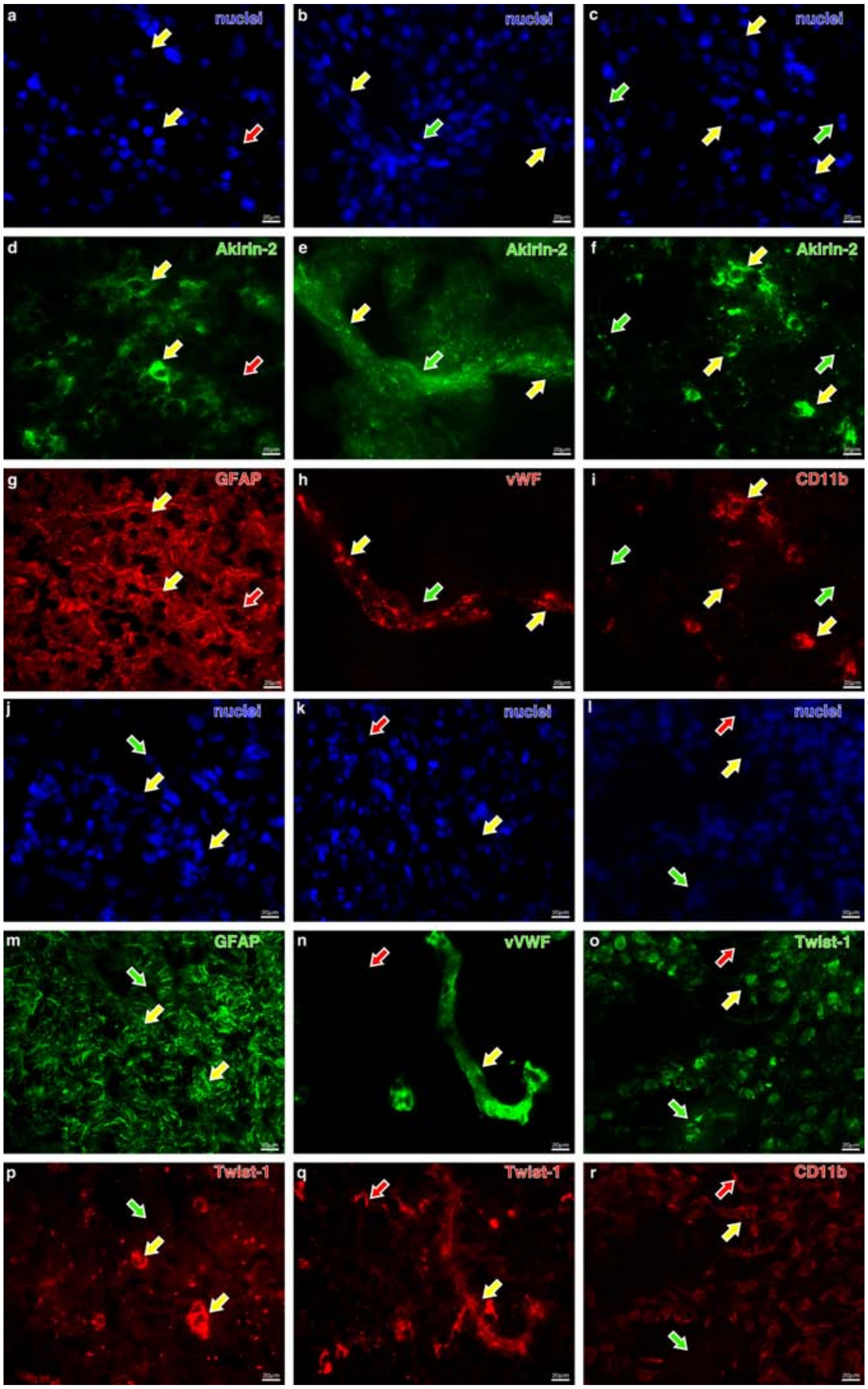

Supplementary Figure S1: Representative costainings of Akirin-2 (a-i) or Twist-1 (j-r) with cellular markers (glial fibrillary acidic protein (GFAP), von Willebrand factor protein (vWF) and CD11b in human solid GBMs as determined by immuno‡uorescence microscopy as supplement to Fig. 2 a-f. The Immunofluorescent stainings are shown as individual images per dye/stained marker: (a-c, j-l) nuclei (blue), (d-f) Akirin-2 (green), (o-q) Twist-1 (green/red), (g-h, m, n, r) GFAP, vWF or CD11b (red). Images in the same column correspond to the respective merged image in Fig. 2 (a, d, g merge to Fig. 2a; b, e, h merge to Fig. 2b; c, f, i merge to Fig. 2c; j, m, p merge to Fig. 2d; k, n, q merge to Fig. 2e; l, o, r merge to Fig. 2f). Arrows indicating single (green or red) or double (yellow) staining of the respective marker are in the same place as in Fig. 2. Magnification 400x, bar: 20 µm, representative examples of three independent experiments are shown.

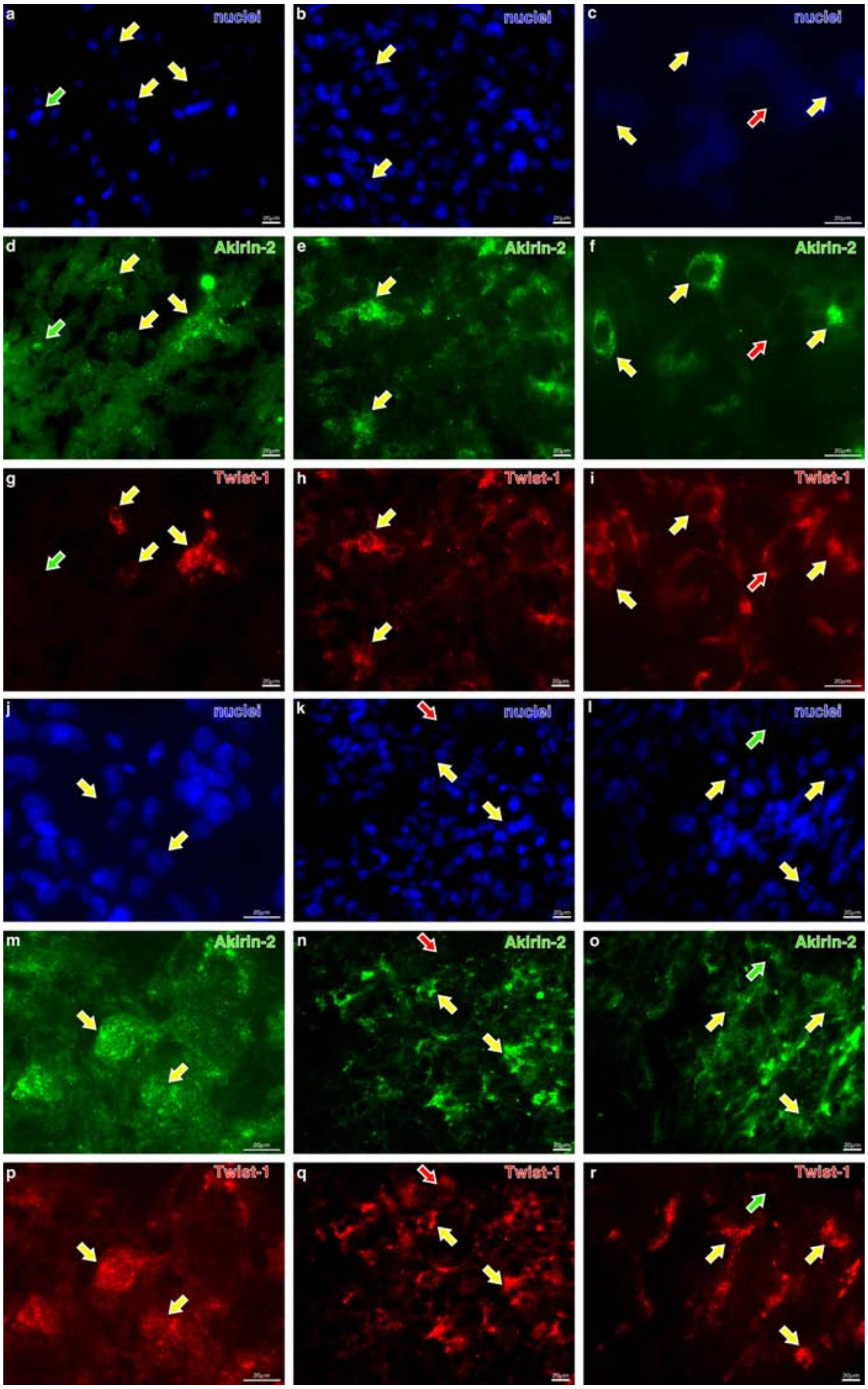

Supplementary Figure S2: Representative costainings of Akirin-2 and Twist-1 in human solid GBMs as determined by immuno‡uorescence microscopy as supplement to Fig. 2 g-l. The Immuno‡uorescent stainings are shown as individual images per dye/stained marker: (a-c, j-l) nuclei (blue), (d-f, m-o) Akirin-2 (green) and (g-I, p-r) Twist-1 (red). Images in the same column correspond to the respective merged image in Fig. 2 (a, d, g merge to Fig. 2g; b, e, h merge to Fig. 2h; c, f, i merge to Fig. 2i; j, m, p merge to Fig. 2j; k, n, q merge to Fig. 2k; l, o, r merge to Fig. 2l). Arrows indicating single (green or red) or double (yellow) staining of the respective marker are in the same place as in Fig. 2. Magniflcation 400x, bar: 20 µm, representative examples of three independent experiments are shown.

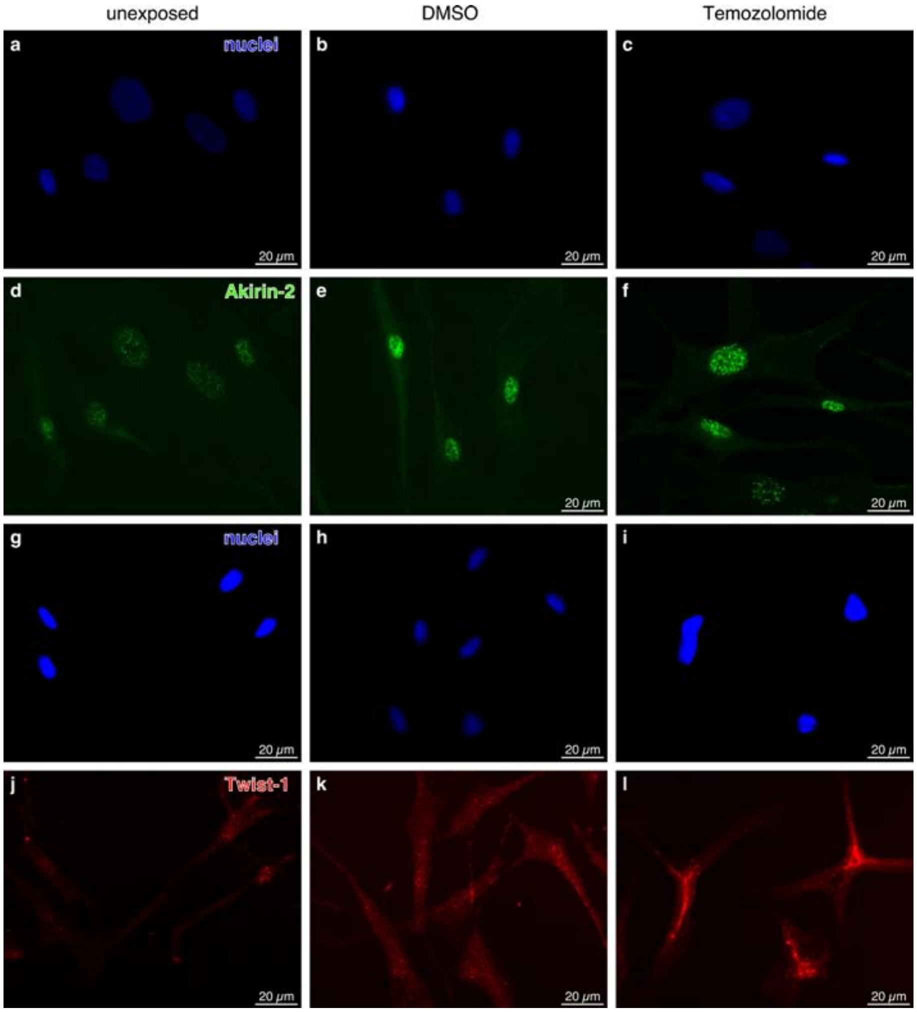

Supplementary Figure S3: Akirin-2 and Twist-1 staining after TMZ treatment in cultured primary human GBM (P3). The Immuno‡uorescent stainings are shown as individual images per dye/marker (as supplement to Fig. 3C a-f): (a-c, g-i) nuclei (blue), (d-f) Akirin-2 (green) and (j-l) Twist-1 (red). Images in the same column correspond to the respective merged image in Fig. 3C (a + d merge to Fig. 3C a; b + e merge to Fig. 3C b; c + f merge to Fig. 3C c; g + j merge to Fig. 3C d; h + k merge to Fig. 3C e; i + l merge to Fig. 3C f). Magnification 400x, bar: 20 µm.

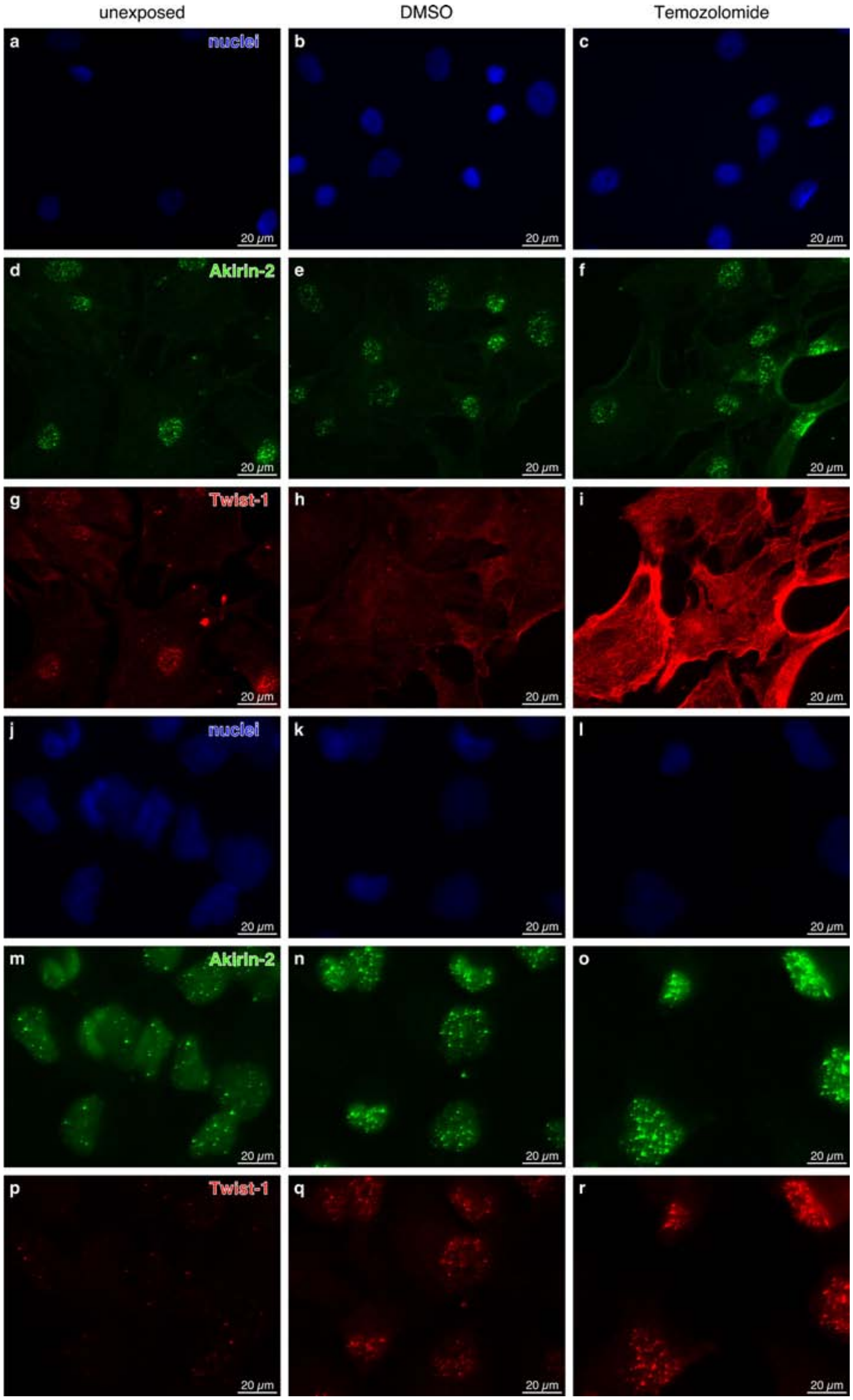

Supplementary Figure S4: Akirin-2 and Twist-1 co-staining after TMZ treatment in cultured primary human GBM (P4) and GBM cell line T98G. The Immuno‡uorescent stainings are shown as individual images per dye/marker (as supplement to Fig. 3C g-l): (a-c, j-l) nuclei (blue), (d-f, m-n) Akirin-2 (green) and (g-I, p-r) Twist-1 (red). Images in the same column correspond to the respective merged image in Fig. 3C (a, d, g merge to Fig. 3C g; b, e, h merge to Fig. 3C h; c, f, i merge to Fig. 3C I; j, m, p merge to Fig. 3C j; k, n, q merge to Fig. 3C k; l, o, r merge to Fig. 3C l). Magniflcation 400x, bar: 20µm, for T98G representative examples of three independent experiments are shown.

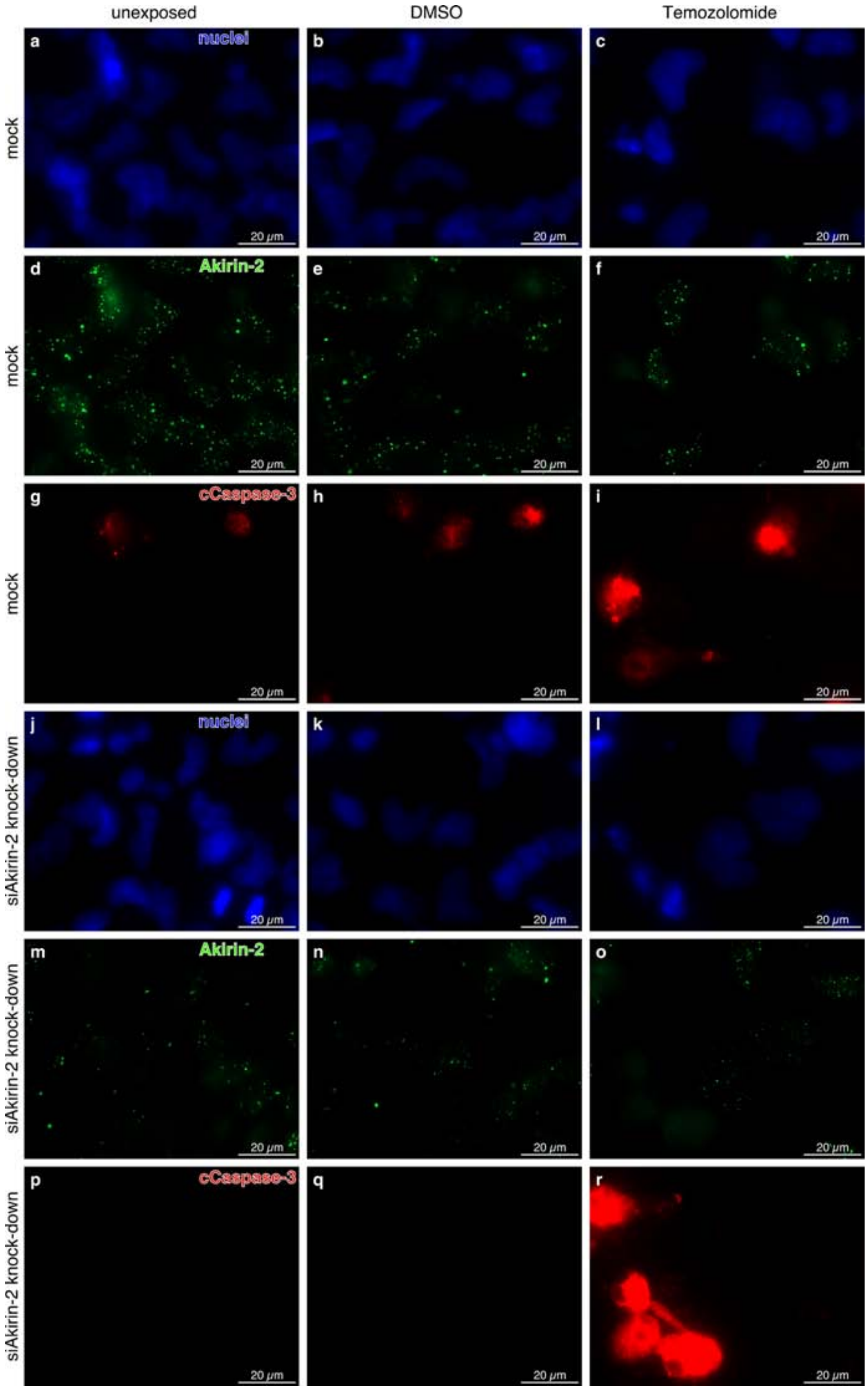

Supplementary Figure S5: Representative costainings of Akirin-2 (green) with cleaved (c)Caspase-3 (red) in unexposed, dimethylsulfoxide (DMSO) or TMZ (400μg/ml, 24 h) treated mock or siAkirin-2 transfected T98G GBM cells. Immuno‡uorescent stainings are shown as individual images per dye/marker (as supplement to Fig. 5 a-f): (a-c, j-l) nuclei (blue), (d-f, m-o) Akirin-2 (green) and (g-i, p-r) cCaspase-3 (red). Images in the same column correspond to the respective merged image in Fig. 5 (a, d, g merge to Fig. 5a; b, e, h merge to Fig. 5b; c, f, i merge to Fig. 5c; j, m, p merge to Fig. 5d; k, n, q merge to Fig. 5e; l, o, r merge to Fig. 5f) Magniflcation 400x, bar: 20μm, representative examples of two independent experiments are shown.

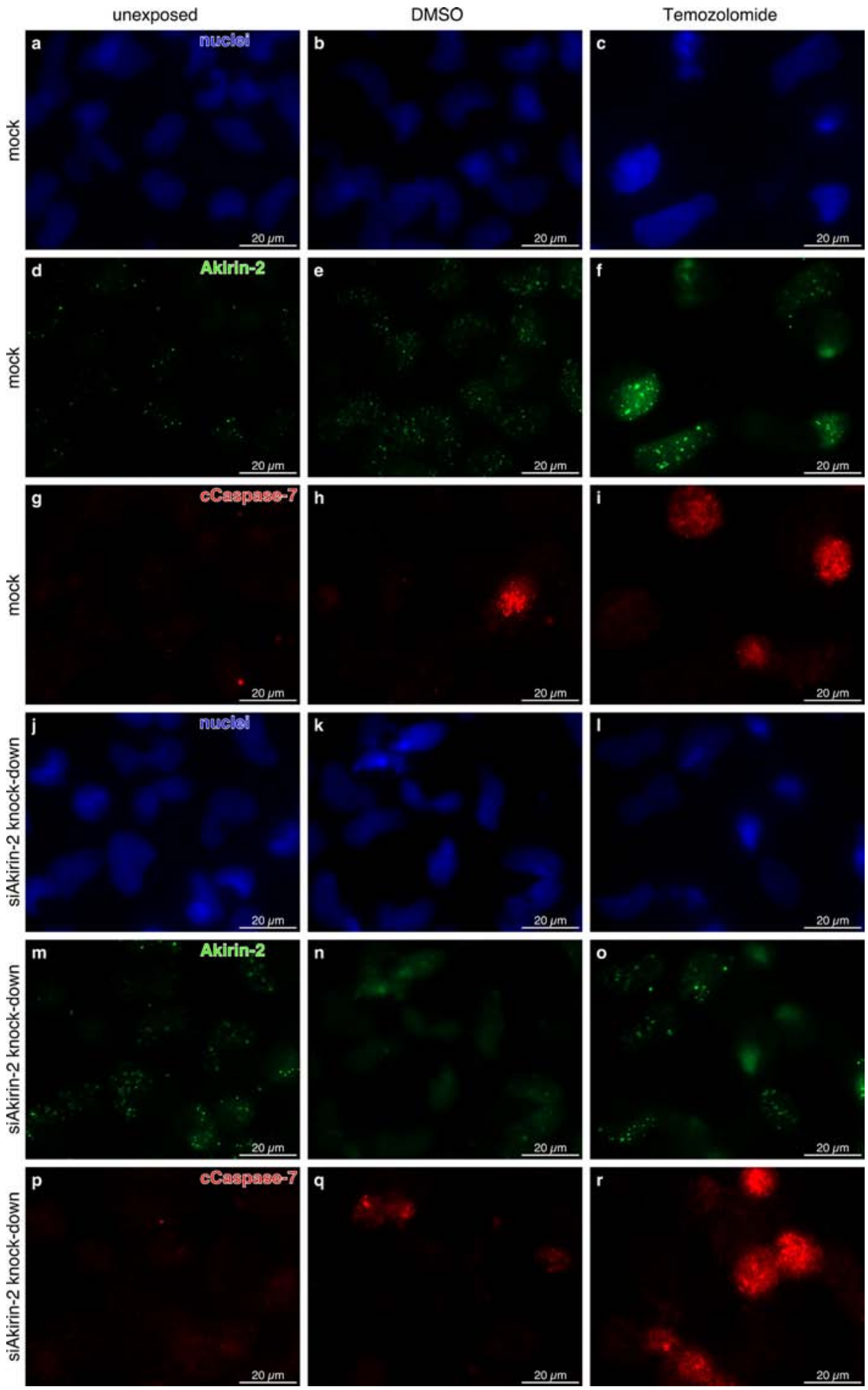

Supplementary Figure S6: Representative costainings of Akirin-2 (green) with cleaved (c)Caspase-7 (red) in unexposed, dimethylsulfoxide (DMSO) or TMZ (400µg/ml, 24 h) treated mock or siAkirin-2 transfected T98G GBM cells. Immuno‡uorescent stainings are shown as individual images per dye/marker (as supplement to Fig. 5 g-l): (a-c, j-l) nuclei (blue), (d-f, m-o) Akirin-2 (green) and (g-i, p-r) cCaspase-3 (red). Images in the same column correspond to the respective merged image in Fig. 5 (a, d, g merge to Fig. 5g; b, e, h merge to Fig. 5h; c, f, i merge to Fig. 5i; j, m, p merge to Fig. 5j; k, n, q merge to Fig. 5k; l, o, r merge to Fig. 5l) Magnification 400x, bar: 20µm, representative examples of two independent experiments are shown.

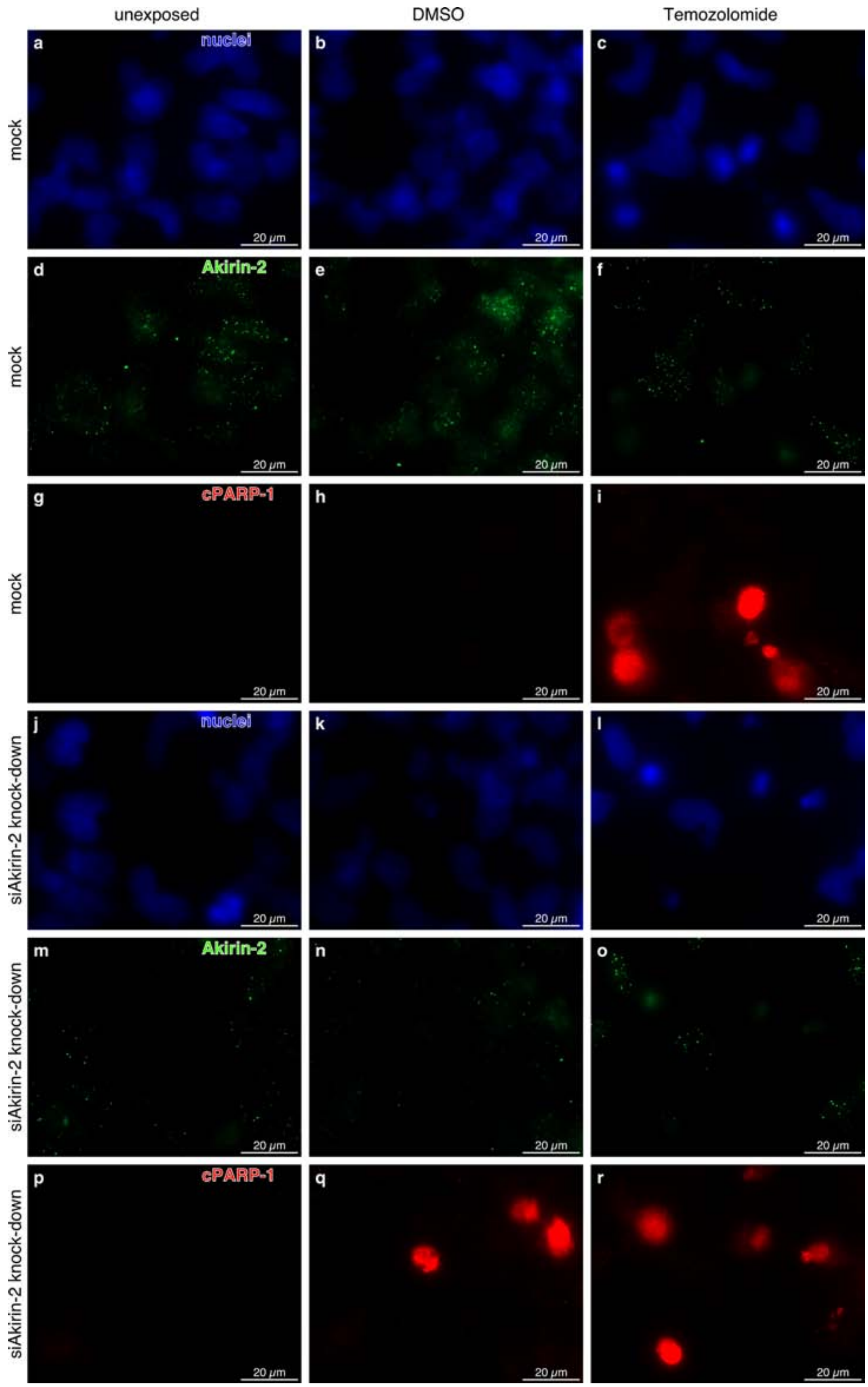

Supplementary Figure S7: Representative costainings of Akirin-2 (green) with cleaved (c)PARP-1 (red) in unexposed, dimethylsulfoxide (DMSO) or TMZ (400µg/ml, 24 h) treated mock or siAkirin-2 transfected T98G GBM cells. Immuno‡uorescent stainings are shown as individual images per dye/marker (as supplement to Fig. 5 m-r): (a-c, j-l) nuclei (blue), (d-f, m-o) Akirin-2 (green) and (g-i, p-r) cCaspase-3 (red). Images in the same column correspond to the respective merged image in Fig. 5 (a, d, g merge to Fig. 5m; b, e, h merge to Fig. 5n; c, f, i merge to Fig. 5o; j, m, p merge to Fig. 5p; k, n, q merge to Fig. 5q; l, o, r merge to Fig. 5r) Magniflcation 400x, bar: 20µm, representative examples of two independent experiments are shown.

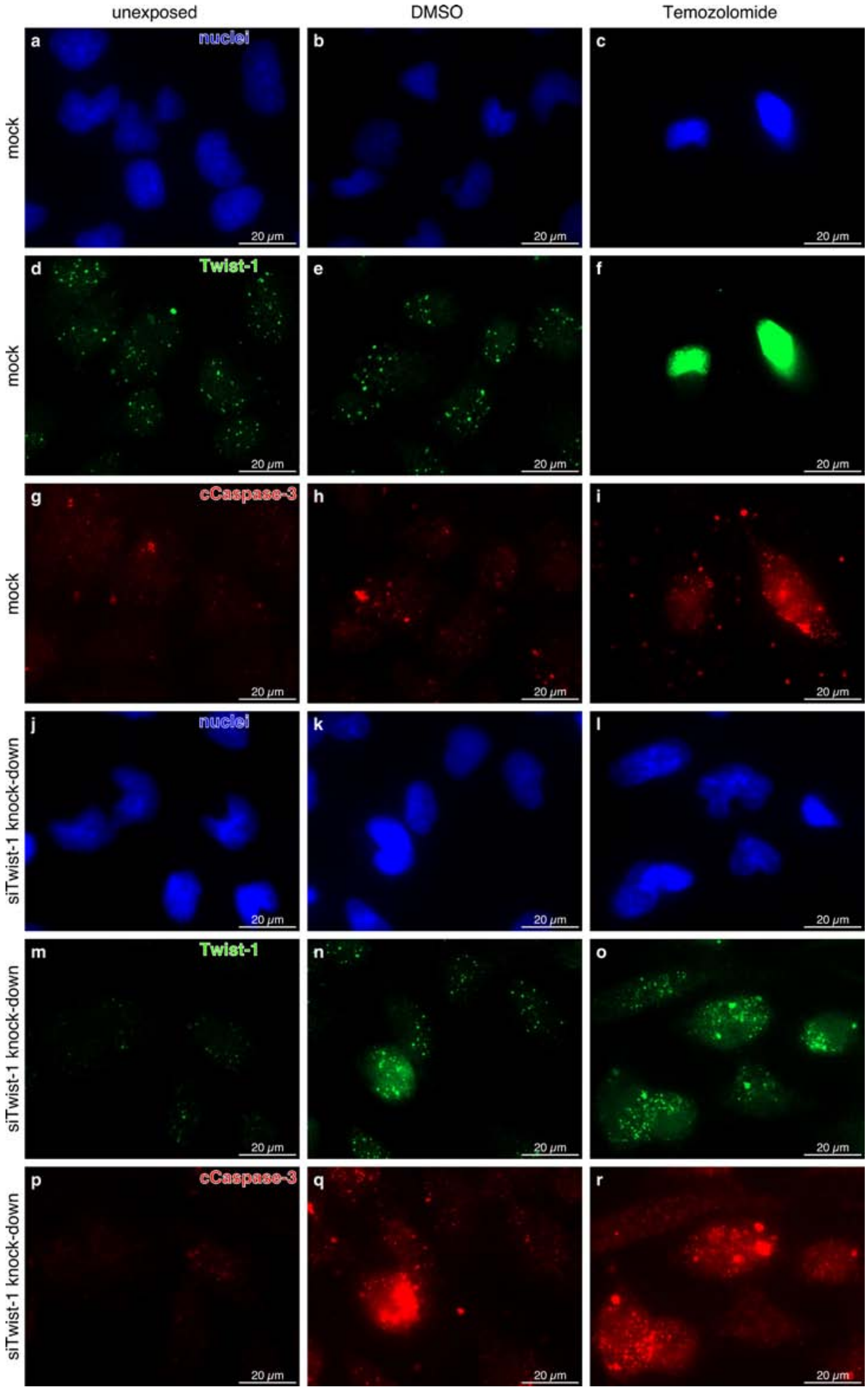

Supplementary Figure S8: Representative costainings of Akirin-2 (green) with cleaved (c)Caspase-3 (red) in unexposed, dimethylsulfoxide (DMSO) or TMZ (400 µg/ml, 24 h) treated mock or siAkirin-2 transfected T98G GBM cells. Immuno‡uorescent stainings are shown as individual images per dye/marker (as supplement to Fig. 6 a-f): (a-c, j-l) nuclei (blue), (d-f, m-o) Akirin-2 (green) and (g-i, p-r) cCaspase-3 (red). Images in the same column correspond to the respective merged image in Fig. 6 (a, d, g merge to Fig. 6a; b, e, h merge to Fig. 6b; c, f, i merge to Fig. 6c; j, m, p merge to Fig. 6d; k, n, q merge to Fig. 6e; l, o, r merge to Fig. 6f) Magnification 400x, bar: 20µm, representative examples of two independent experiments are shown.

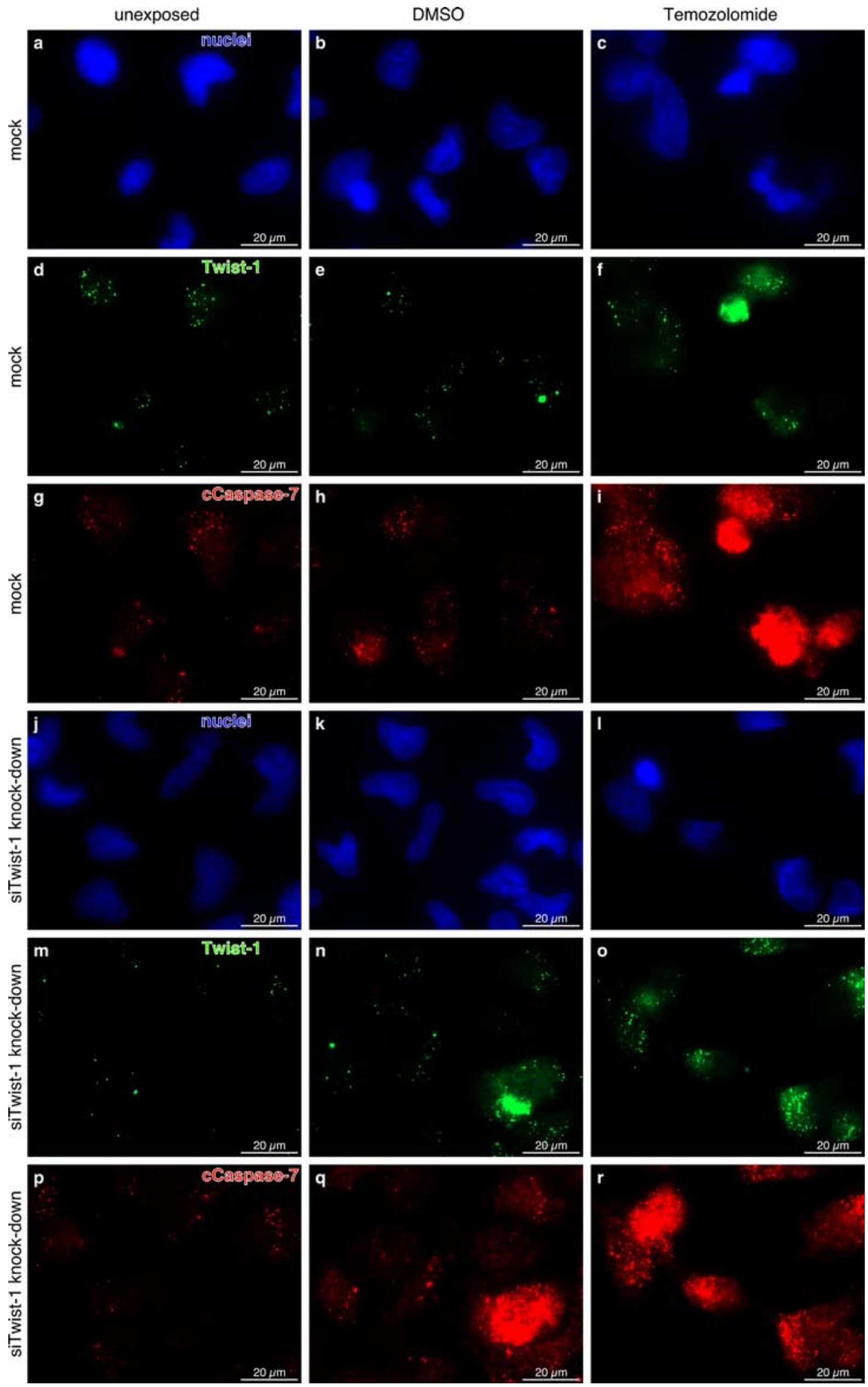

Supplementary Figure S9: Representative costainings of Akirin-2 (green) with cleaved (c)Caspase-7 (red) in unexposed, dimethylsulfoxide (DMSO) or TMZ (400μg/ml, 24 h) treated mock or siAkirin-2 transfected T98G GBM cells. Immuno‡uorescent stainings are shown as individual images per dye/marker (as supplement to Fig. 6 g-l): (a-c, j-l) nuclei (blue), (d-f, m-o) Akirin-2 (green) and (g-i, p-r) cCaspase-3 (red). Images in the same column correspond to the respective merged image in Fig. 6 (a, d, g merge to Fig. 6g; b, e, h merge to Fig. 6h; c, f, i merge to Fig. 6i; j, m, p merge to Fig. 6j; k, n, q merge to Fig. 6k; l, o, r merge to Fig. 6l) Magnification 400x, bar: 20μm, representative examples of two independent experiments are shown.

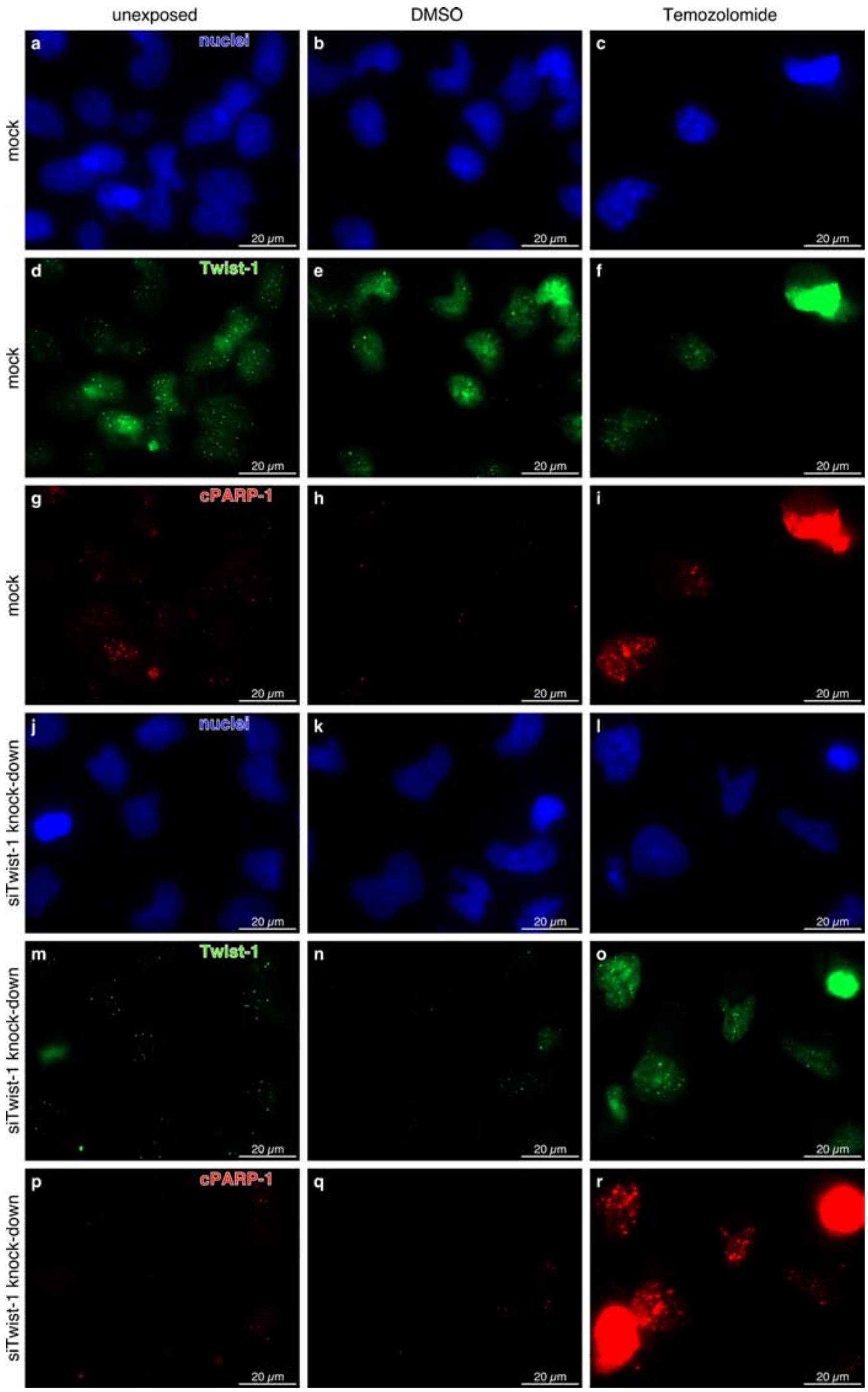

Supplementary Figure S10: **Representative costainings of Akirin-2 (green) with cleaved (c)PARP-1 (red) in unexposed, dimethylsulfoxide (DMSO) or TMZ (400µg/ml, 24 h) treated mock or siAkirin-2 transfected T98G GBM cells. Immuno‡uorescent stainings are shown as individual images per dye/marker (as supplement to Fig. 6 m-r): (a-c, j-l) nuclei (blue), (d-f, m-o) Akirin-2 (green) and (g-i, p-r) cCaspase-3 (red). Images in the same column correspond to the respective merged image in Fig. 6 (a, d, g merge to Fig. 6m; b, e, h merge to Fig. 6n; c, f, i merge to Fig. 6o; j, m, p merge to Fig. 6p; k, n, q merge to Fig. 6q; l, o, r merge to Fig. 6r) Magnification 400x, bar: 20 µm, representative examples of two independent experiments are shown.**
